# Synthesis of Pyrazolone Derivatives and Their Nanometer Ag(I) Complexes and Physicochemical, DNA Binding, Antitumor, and Theoretical Implementations

**DOI:** 10.1155/2018/2727619

**Published:** 2018-05-14

**Authors:** Ismail Althagafi, Nashwa M. El-Metwaly, Marwa G. Elghalban, Thoraya A. Farghaly, Abdalla M. Khedr

**Affiliations:** ^1^Chemistry Department, College of Applied Sciences, Umm Al-Qura University, Mecca, Saudi Arabia; ^2^Chemistry Department, Faculty of Science, Mansoura University, Mansoura, Egypt; ^3^Department of Chemistry, Faculty of Science, University of Cairo, Giza 12613, Egypt; ^4^Chemistry Department, Faculty of Science, Tanta University, Tanta, Egypt

## Abstract

Four pyrazolone derivatives and their corresponding silver complexes were synthesized and characterized. Based on elemental analysis, 1 : 2 (M : L) molar ratio was suggested for all inspected complexes. ^1^H, ^13^C NMR, mass, UV-Vis, TGA, and IR were the spectral tools used for describing the formulae. Moreover, XRD patterns and SEM pictures were used to evaluate the particle sizes which appeared strongly in nanometer range. CT-DNA study is the major consideration in this study, to test the interacting ability of all synthesized cationic complexes towards cell DNA. Each binding constant was computed and correlated with the Hammett sigma constant. Antitumor activity was examined upon three carcinoma cell lines (MCF-7, HepG2, and HCT116). The high efficiency was recorded towards MCF-7 (breast carcinoma) cell line. Kinetic studies yield essential parameters to assert on the rule of metal atom on thermal feature of organic compounds. Molecular modeling was implemented to optimize the structures of compounds. Also, molecular docking was achieved to obtain a clear view about proposed drug behavior within the affected cells. This was achieved through comparing the calculated internal energy values of all docking complexes. All the tested compounds displayed a significant interaction with breast cancer protein (strong matching with practical result) followed by DNA polymerase protein.

## 1. Introduction

After discovering the cisplatin as an effective antitumor drug, the interest of researchers in the coordination chemistry increased to design highly effective complexes as antiantitumor operators [1–3]. But, the limitations of the clinical success of cisplatin due to critical side effects and resistance that cause retrogression [4] have stimulated searches for other metal complexes. The synthesis for series silver(I) complexes as antitumor agents conquers a considerable place in cancer chemotherapy, as evidenced by their remarkable therapeutic potential reflected in many reported research works [5]. The silver ion is of considerable solicitude in the design of coordination complexes due to the resilience of its coordination sphere and the ease with which it varies its coordination number (from 2 to 4, rarely 5 or 6). Coordination complexes of silver are known to be convertible through changes in ligand geometry, rigidity or functionality, and/or by modifications to the counter-ion or solvent system [6]. But, silver often tends to prepare coordination polymers [7, 8]. The main factor in the effectiveness of anti-cancer compounds is their ability to interact with the DNA of the cancer cell which prevents its division until the cell will die [9]. Ag^+^ is uniquely interesting because it binds exclusively to the bases rather than the backbone of DNA [10, 11]. On the other hand, the wide range of biological activities of pyrazolone as antitumor [12], anti-inflammatory [13] and antimicrobial [14] makes it a basic objective to synthesize biologically active transition metal complexes as it is known that the formation of the complexes enhances the biological efficiency of the ligand [15]. Based on all of the afore-mention, we focused in this work on the synthesis of nonpolymeric silver complexes with pyrazolone to improve its efficiency as anticancer drug for three carcinoma cell lines and to study their interaction with DNA. The structure of novel complexes will be investigated through all possible spectral and analytical tools. Computational studies (i.e., Gaussian 09 molecular modeling, QSAR, and Autodock tools) will be applied to prove and interpret the efficiency of novel complexes.

## 2. Experimental Work

### 2.1. Chemical Reagents

All chemicals used to prepare pyrazolone derivatives (ethyl cyanoacetate, aniline, 4-chloroaniline, 4-toluidine, 4-nitroaniline, hydrazine hydrate, ethanol, sodium acetate trihydrate, sodium nitrite, and HCl) were used as received without any pretreatment. They were purchased from Fluka and Sigma-Aldrich. Also, AgNO_3_ used for preparing the complexes was PDH.

### 2.2. Preparations

#### 2.2.1. Synthesis of 3-Amino-4-(2-substituted phenylhydrazono)-1-*H*-pyrazol-5(*4H*)-one

**Scheme 1:**
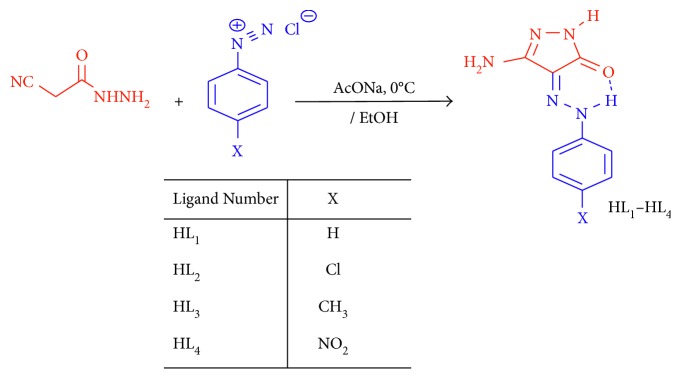
Synthesis of pyrazolone ligands (HL^1^–HL^4^).

**Figure 1:**
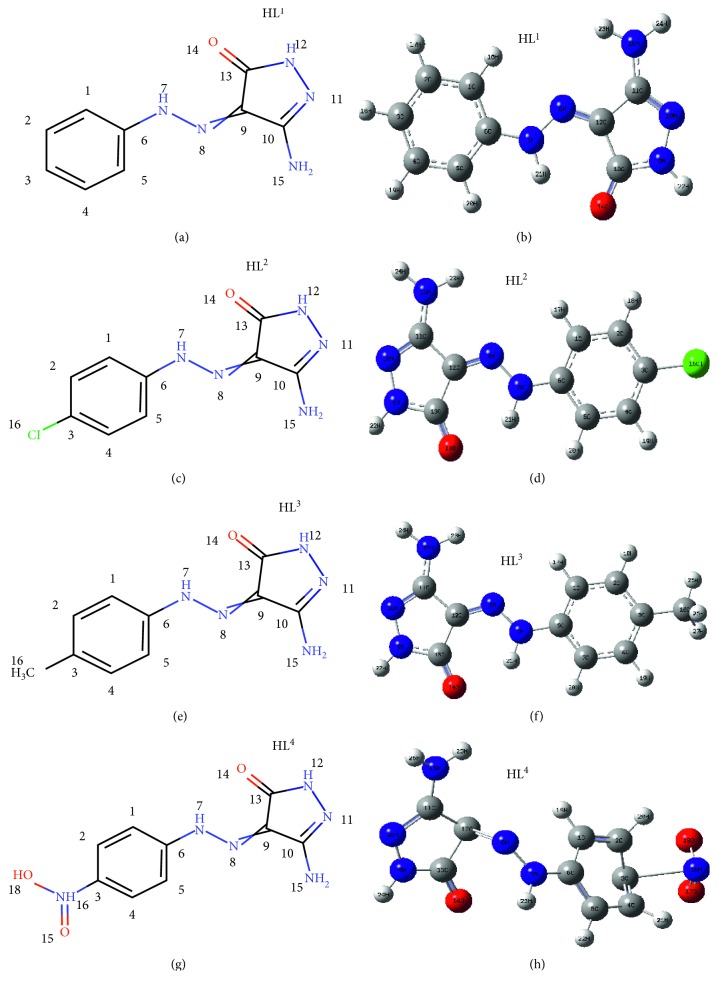
The best structural forms of pyrazolone ligands (HL^1^–HL^4^).

**Figure 2:**
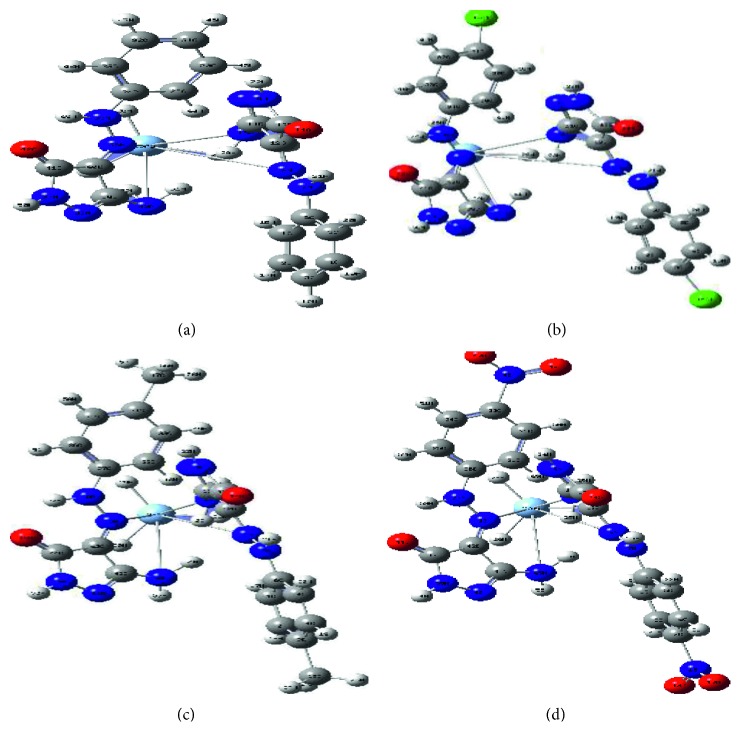
Optimized structures for Ag(I)-HL (1–4) complexes (a–d, respectively).

Derivatives of pyrazolone (HL^1–4^) were synthesized and fully characterized in accordance with the previous studies [16, 17]. A fresh prepared solution of cyanoacetic hydrazide (0.99 g, 0.01 mol) in ethanol (50 ml) was coupled in presence of sodium acetate trihydrate (1.36 g, 0.01 mol). The coupling was carried out on 0.01 mol arenediazonium chloride (prepared by diazotizing corresponding aniline derivative) in 6 M hydrochloric acid (6 ml) with cold sodium nitrite solution (10 ml, 1 M; as known) in ice bath (at 0–5°C). The reaction mixture was set in a refrigerator for 3 hrs. The precipitating compound was filtered off and dried. Refluxing ethanolic arylhydrazone solution for 2 hr gives the aimed ligands, 3-amino-4-(2-substituted phenylhydrazono)-4,5-dihydropyrazol-5-ones (HL^1–4^) as appeared in Scheme 1. Whereas, their scientific names are HL^1^, 3-amino-4-(2-phenylhydrazono)-1*H*-pyrazol-5(*4H*)-one; HL^2^, 3-amino-4-(2-(4-chlorophenyl)hydrazono)-1*H*-pyrazol-5(*4H*)-one; HL^3^, 3-amino-4-(2-(4-mehylphenyl)hydrazono)-1*H*-pyrazol-5(*4H*)-one and HL^4^, 3-amino-4-(2-(4-nitrophenyl)hydrazono)-1*H*-pyrazol-5(*4H*)-one. The prepared pyrazolone compounds were structurally optimized to clarify the orientation of active groups (Figure 1) using Gaussian 09 software. ^1^H NMR (DMSO-*d*_6_) spectra of four ligands (Figure 1S) were assigned as follows: HL^1^, *δ*: 5.80 (s, 2H, NH_2_), 7.10–7.54 (m, 6H, Ar–H, and amidic-NH), 10.53 (s, 1H, NH). HL^2^, *δ*: 5.84 (s, 2H, NH_2_), 7.42–7.59 (m, 5H, Ar–H and amidic-NH), 10.53 (s, 1H, NH). HL^3^, *δ*: 2.23 (s, 3H, CH_3_), 5.71 (s, 2H, NH_2_), 7.01–7.60 (m, 5H, Ar–H and amidic-NH), 11.41 (s, 1H, NH), and HL^4^, *δ*: 6.02 (s, 2H, NH_2_), 7.73–8.25 (m, 5H, Ar–H and amidic-NH), 10.66 (s, 1H, NH). Also, mass spectral analysis of HL^2^ and HL^3^ ligands (Figure 2S) displays the following fragmentation peaks starting with molecular ion; *m*/*z* = 239 (M^+^ + 2, 21.76); 238 (M^+^ + 1, 8.61); 237 (M^+^, 64.69); 126 (100); 111 (26.80); 98 (36.92); 91 (9.09) and 218 (M^+^ + 1, 13); 217 (M^+^, 100); 126 (39.26); 111 (1.32); 106 (38.82); 77 (46.96), respectively.

#### 2.2.2. Synthesis of Silver(I) Complexes

2 mmol AgNO_3_ was dissolved in a little amount of ethanol. Then, the solution was added gradually to 2 mmol from each ligand solution (in DMF). The mixture was stirred and refluxed at 50–60°C for 15–30 min. The complexes appeared in the reaction medium without external additions. The main reaction product was precipitated, then filtered off and washed with ethanol followed by diethyl ether, and after that, dried over CaCl_2_ desiccators.

### 2.3. DNA Binding Methodology

The binding efficiency of calf thymus-DNA (CT-DNA) has been deliberated by spectrophotometric titration method. 50 mg from CT-DNA were dissolved by continuous stirring overnight in deionized water (pH = 7.0) and preserved at 4°C. Tris(hydroxymethyl)-aminomethane was used to prepare 5.0 mM tris-HCl buffer (pH = 7.2) after adding 50 mM NaCl in deionized water. The prepared buffering solution is perfectly free from protein, based on the value measured for absorbance ratio (1.8–1.9; A260/A280) at 260 and 280 nm [18]. The concentration of prepared DNA (5.25 × 10^−4^ M) was computed by knowing molar absorptivity coefficient which is 6600 M^−1^·cm^−1^ at 260 nm. 2.5 × 10^–5^ M was the concentration used from each complex. At room temperature, the UV-Vis scanning process was carried out at the fixed range (200–900 nm) by 1 cm quartz cuvette. A gradual increment for CT-DNA concentration from 0.00 till 1.58 × 10^−4^ mol·L^–1^ was done, towards a fixed concentration from each tested compound. The same CT-DNA amount was added to a reference buffer solution with each measurement, to omit the absorbance of free DNA. Intrinsic binding constant (*K*_b_) of the interaction between compound and CT-DNA was computed by; [DNA]/(*єa* − *єf*) = [DNA]/(*єb* − *єf*) + 1/*K*_b_ (*єa* − *єf*), equation [19]. Where, [DNA] is the molar concentration from DNA, *єa* is the extinction coefficient observed at each DNA concentration by; *A*_obs_/[compound], *єf* is the extinction coefficient for each free tested compound in solution. Moreover, *єb* is the extinction coefficient for the fully bonded compound towards DNA. By drawing the relation of [DNA]/(*єa* − *єf*) versus [DNA], *K*_b_ is given by the following ratio: slope/intercept.

### 2.4. In Vitro Antitumor Study

The biological feature of synthesized pyrazolone derivatives (HL^1–4^) and their Ag(I) complexes were screened towards three carcinoma cell lines (MCF-7, HEPG-2, and HCT-116) by sulforhodamine B (SRB). Cells were kept in RPMI medium enriched with 100 *µ*g/ml streptomycin, 100 units/ml penicillin, and 10% heat-inactivated genetic bovine serum inhibit, 5% (v/v) CO_2_ atmosphere at 37°C, and cells were subcultured two times/week. The growing cells were gathered using trypsin–EDTA (0.25%) and plated in 96-well plates at 1000 cells/well. Cells were treated to extraction for 72 h and subsequently fixed with TCA (10%) for 1 h at 4°C. After washing times, cells were exposed to SRB solution (0.4%) for 10 min in a dark place and subsequently washed with glacial acetic acid (1%). After keeping overnight, Tris–HCl was used to dissolve SRB-stained cells, and the intensity of color was measured to be 540 nm. The viability of cell was measured by Trypan blue (0.4) stain, which permits us to differentiate viable (nonstained) from dead (stained) cells. Viable and dead cells were counted with Nikon microscope implementing hemocytometer. Surviving cells percentage = (1.00 − (number of blue cells/number of total cells)) × 100. Statistical analysis was executed by using Sigma Plot version 12.0.

### 2.5. Equipments and Physical Analysis

Carbon, hydrogen, and nitrogen contents were performed at Perkin-Elmer 2400 CHN elemental analyzer. The silver content was evaluated gravimetrically using standard method [20]. The molar conductivities of metal complexes were obtained by JENWAY model 4070 conductance bridge (in DMSO solvent). KBr-IR and ^13^C, ^1^H NMR spectra were obtained by JASCO FT-IR-4100 spectrophotometer (400–4000 cm^−1^) and Burker 500 MHz, respectively. The purity of most compounds was checked from mass spectra at 70 eV by using AEIMS 30 mass spectrometer with heating rate 40°C/min and mass range (50–1000). Electronic spectra were recorded by UV_2_ Unicam UV/Vis spectrophotometer at room temperature using 1 cm quartz cell (in DMSO solvent). Thermal analysis was accomplished using Shimadzu thermogravimetric analyzer by heating rate of 10°C min^−1^ under nitrogen (20–900°C). X-ray diffraction patterns (XRD) were accomplished by Rigaku diffractometer using Cu/K*α* radiation. Scanning electron microscopy (SEM) images were extracted by the Joel JSM-6390 equipment. Antitumor activity was tested in microbiology center.

### 2.6. Computational Implementation

#### 2.6.1. Kinetics

Essential thermodynamic parameters were extracted over two degradation stages from TGA curves. All degradation processes were carried out at a satisfactory rate which introduces a good computation for kinetic parameters along most degradation stages. The kinetic parameters were calculated for the first and second degradation stages. The chosen steps exhibit the decomposition for great masses from the coordination sphere, and order (*n*) and energy of activation (*E*) were computed. Many researches were dealing with kinetic calculations [21–29] and proposed equations stated for such purpose. Among these methods, Coats and Redfern [23] and Horowitz and Metzger [28]were used in this study.

#### 2.6.2. Structural Optimization

Utilizing Gaussian 09 software [30], the structural optimization for pyrazolone derivatives (HL^1–4^) and their Ag(I) complexes were executed. DFT/B3LYP is a proper method achieving best configurations and computational files. The files extracted from the optimization process (log and chk files) were visualized by Gauss-View screen [31] to produce essential parameters. Also, HOMO and LUMO images were extracted from chk file visualization.

#### 2.6.3. QSAR Calculation

QSAR parameters calculations were carried out over all synthesized compounds after optimization process by applying HyperChem software program (8.1). Firstly, preoptimization was accomplished by soft adjustment procedures, through molecular mechanics force field (MM^+^) followed by semiempirical (AM1). The program implemented over structural forms without fixing any parameter to execute the optimization at equilibrium. The minimization of energy was done by Polak–Ribière conjugated gradient algorithm. QSAR parameters were computed for all derivates and their Ag(I) complexes, for comparison.

#### 2.6.4. Molecular Docking Methodology

The docking process was executed between protein receptors (3s7s, 3e1r, and 4dk7 assign for breast, colon, and liver cancers, respectively) and pyrazolone derivatives (HL^1–4^) (proposed inhibitors). Moreover, the docking process was done over CT-DNA polymerase (1bpb). The selected protein receptors were the same for the pathogen of tumor cells investigated in the experimental part to assert on the results. Autodock tools 4.2 is a software used for docking methodology. Gasteiger partial charges were added over elements of tested compounds (pyrazolones). The rotatable bonds were omitted and nonpolar hydrogen atoms were ruling out. The energy levels of docked complexes were computed after addition of fundamental hydrogen atoms, Kollman united atom type charges, and salvation parameters [32]. Affinity (grid) maps of ××Å grid points and 0.375 Å spacing were generated utilizing Autogrid program [33]. Also, Vander Waal forces and electrostatic terms were acquired, through the use of autodock parameter set-dependent and distance-dependent dielectric functions, respectively. The docking was accomplished using Solis and Wets local search method and Lamarckian Genetic algorithm (LGA) [34]. Initial position, orientation, and torsions of tested molecule (inhibitor) were set indiscriminately. All rotatable torsions were expelled during the docking process. Each experiment is the average value for 10 different runs that were set to close after maximum of 250000 energy assessments. 150 is the used population size. During the process, translational step of 0.2 Å, quaternion, and torsion steps of 5 were used.

## 3. Results and Discussion

### 3.1. Physicochemical Properties

Common specific features are aggregated in Table 1. All synthesized complexes have high melting points (˃300°C), while that of free organic derivatives (HL^1–4^) were 246, 300, 252, and 300°C. The analytical results are proper with 1 : 2 molar ratio between silver ion and chelating ligands. The molar conductivity (in DMSO) which measured for all complexes (1 mmol) is coinciding with conductivity feature. The conductivity values (Λ_m_) are confined in the range of conducting feature for monoanionic conjugated nitrate with cationic coordination sphere [35]. This is proper feature with high acidic anions as nitrate.

### 3.2. IR and ^1^H, ^13^C NMR Assignments

**Figure 3:**
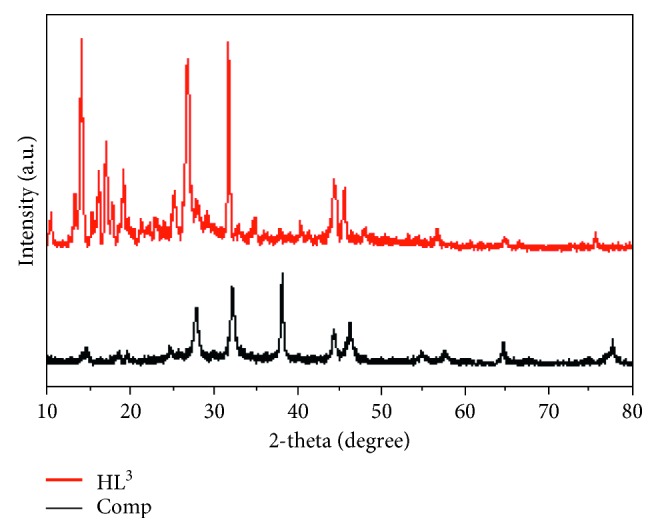
X-ray diffraction pattern of pyrazolone, HL^3^, and its AgNPs complex.

IR spectral analysis of pyrazolone (HL^1–4^) derivatives (Table 1S) displays significant bands assigned for the following vibrations; *ν*_as_(NH_2_), *ν*_s_(NH_2_), *ν*(NHs), *δ*(NH_2_), *δ*(NH), *ν*(C=N), and *ν*(C=O). Observable lower shifted appearance for *ν*(NH_2_), *δ*(NH_2_), and *ν*(C=N) bands suggests the bidentate contribution mode of pyrazolones towards silver atom. However, more or less shifted appearance of NHs and C=O donors introduces their ruling out from coordination [36]. Each coordination sphere includes two coordinating ligands towards one central atom, in which the orientation of donors according to central atom is the cause for chelating mode (by NH_2_ and C=N)and also has the major impact for ruling out of other donors (NHs and C=O). The appearance of new band at ≈1383 cm^−1^ refers to *ν*(NO_3_) about the ionic attachment of nitrate. Moreover, new band appeared at the range 509–560 cm^−1^ in all complexes, refers to *ν*(M–N) vibration [37–39]. ^1^H and ^13^C NMR (DMSO-*d*_6_) spectral data of investigated complexes are tabulated (Table 2). ^1^H NMR spectra (Figure 3S) display a considerable down field shift for NH_2_ group by an observable ppm value, whereas ^13^C NMR spectral data represent the absence of significant alteration in hybridization attitude upon carbon atoms inside the forms. This is omitting hydrogen relocation through tautomerism followed by the enolization process.

### 3.3. Electronic Spectra

The preparation of AgNPs has been assured through UV-Vis spectral analysis, which carried out for all complexes in the DMSO solvent (10^−3^ M). The measurements process was accomplished at 510, 440, 420, and 480 nm for four Ag(I)-HL (1–4) complexes, respectively. The excessive presence for AgNPs was expected from the deep color of Ag(I) complexes. The dark color of complex solution points to the formation of AgNPs. The electronic spectra of four complexes reflect a well-defined Plasmon band between 420 and 510 nm in the nanoscale range, according to silver nanoparticles association. According to theoretical and experimental studies [40], the optical features of AgNPs were reported based on electronic spectral band position. The band at 420 nm for Ag(I)-HL^3^ complex solution is referring to the presence of small spherical silver nanoparticles. The spherical particles display *λ*_max_ band at shorter wave length, while the nonspherical shape of particles shifts the *λ*_max_ to longer wave length [41]. The higher the intensity of the complex color, the higher the concentration of AgNPs [42]. The tetrahedral configuration of Ag(I) complexes (Figure 2) was proposed based on analytical, IR, ^1^H, ^13^C NMR, and mass spectral analysis, which is acceptable for *d* ^10^ systems.

### 3.4. Microanalysis Mass Spectra

**Scheme 2:**
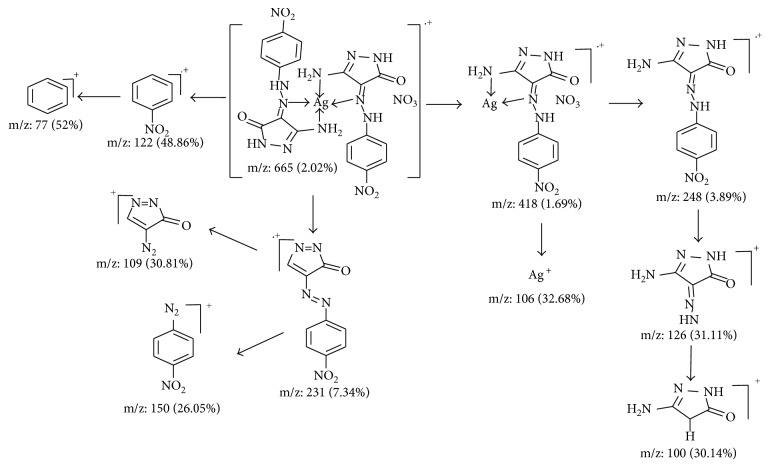
The fragmentation perception for [Ag(HL^4^)_2_](NO_3_) complex.

**Figure 4:**
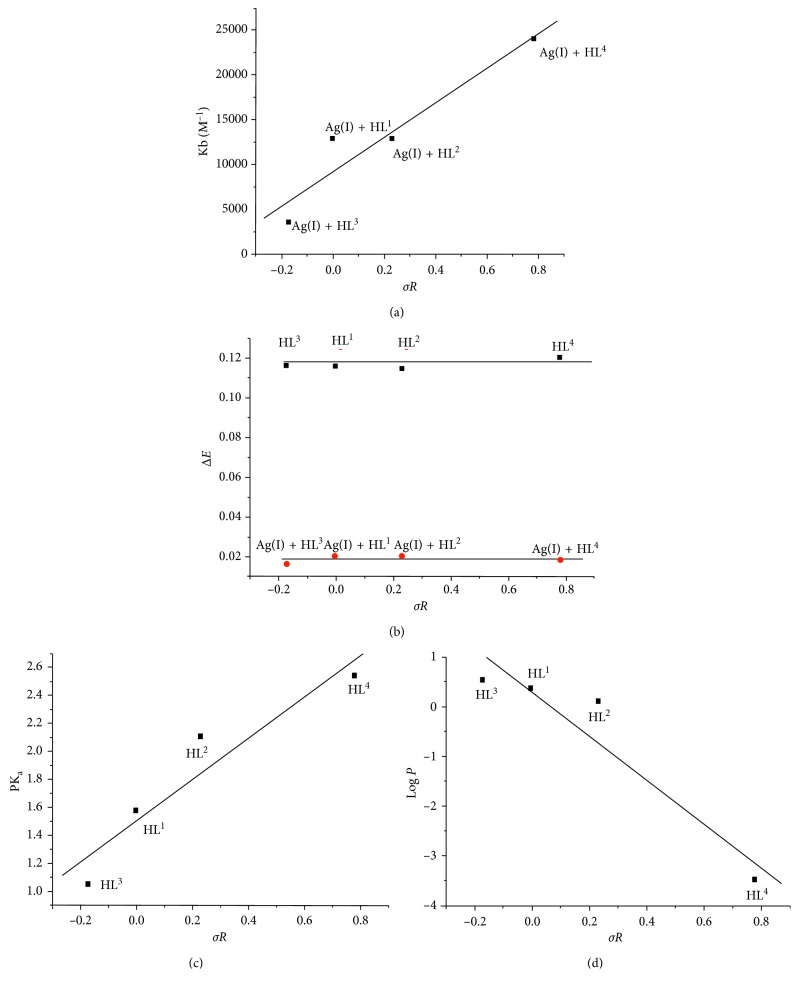
Hammett's relation between effect of *p*-substituent (*σR*) versus Δ*E*, PK_a_ (docking parameter), log *P*, and intrinsic binding constants (*K*_b_) of Ag(I)-HL^1–4^ complexes.

Two organic ligands were chosen for this study (HL^2^ and HL^3^), the molecular ion peaks recorded were *m*/*z* = 239 (M^+^ + 2, calcd. 237.63) and 218 (M^+^ + 1, calcd. 217.23), respectively. A high agreement between each recorded M^+^ and the calculated one supports high purity. Three silver complexes were studied to assert on the formulae proposed (Figure 4S). In the [Ag(HL^2^)_2_](NO_3_) complex spectrum, a well-defined parent peak was appeared at *m*/*z* = 647.66 (M^+^ + 2 (1.2), calcd. 645.15) is followed by a successive fragmentation corresponding to peaks at 344 (3.81), 243 (11.78), 238 (7.55), 220 (14.50), 197 (81.93), 140 (15.61), 127 (39.25), 112 (7.27), 104 (16.64), 95 (15.05), and 95 (65.29). Also, in the [Ag(HL^3^)_2_](NO_3_)2H_2_O complex spectrum, a significant ion peak was recorded at *m*/*z* = 605 (25.94, calcd. 640.36) which is attributing to M^+^ + 1 − (2H_2_O). This is not considered a strange behavior; the sudden expel for water molecules may happen during vaporization. The vaporization is a prefragmentation process before electron bombardment [43]. Essential fragmentation peaks recorded are 546 (54.26), 386 (5.28), 370 (100), 268 (12.45), 243 (19.73), and 202 (1.06). Moreover, in the [Ag(HL^4^)_2_](NO_3_) complex spectrum, the recorded molecular ion peak attributes to M^+^ − 1 (*m*/*z* = 665.22) (calcd. 666.27). The more proper fragmentation perception was displayed in Scheme 2.

### 3.5. Thermogravimetric Analysis

A proper degradation path for all pyrazolone derivatives and their corresponding silver complexes is tabulated (Table 2S). The TGA curves of pyrazolone ligands display two significant degradation stages starting from ≈150°C and ended to ≈620°C. The successive degradation process had three to four stages (ended at ≈800°C). All the coordination spheres around the silver atom were completely destroyed along the temperature range applied. Moreover, the residue includes AgN_3_ or silver atom meanwhile polluted by carbons. A satisfactory agreement between calculated and found weight loss corresponding to the expelled particles reflects the exact assignments for step borders, which suggests that well computed kinetics may be obtained.

### 3.6. XRD and SEM Analysis

**Figure 5:**
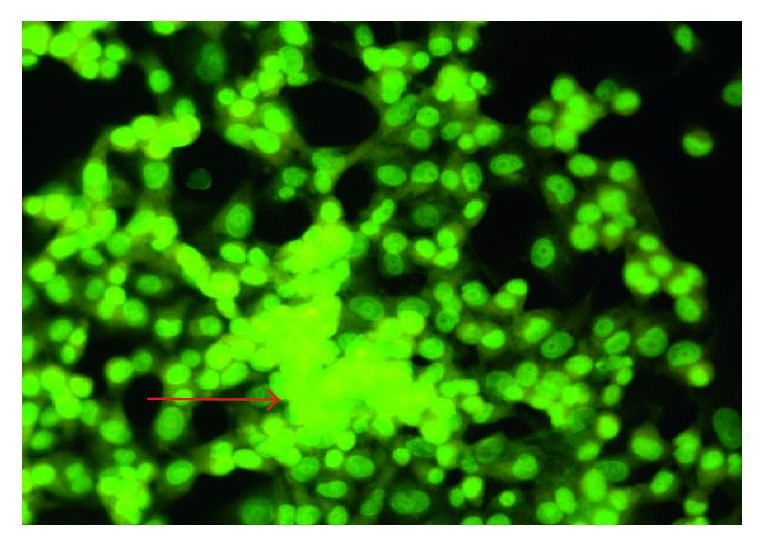
The arrow appeared is attributing to the foci of MCF-7 despite IC_50_ of variable compounds.

XRD patterns of pyrazolone ligands and their silver complexes (solid samples) were recorded (Figures 3 and 5S). This analysis gives a good insight about the crystal lattice for tested compounds. Applying referenced methods, the purity of each compound can be asserted through comparative study with the reactants patterns [44]. Moreover, essential lattice parameters were deducted from high intense peak in patterns (FWHM). All tested compounds show nanocrystalline feature, which clearly impacted on the sharpness of patterns peaks. This may be due to the fixation of compounds' nucleus, as the ligands family and silver central atom. Particle sizes, 2*θ*, relative intensity (%), *d* spacing, and FWHM are represented in Table 3. The sizes of crystals were estimated from the Deby–Scherrer equation: *β* = 0.94*λ*/(*S* cos *θ*), where *S* is the crystallite size, *θ* is the angle of diffraction, and *β* is FWHM, Cu/K*α* (*λ*) = 1.5406 Å.

**Figure 6:**
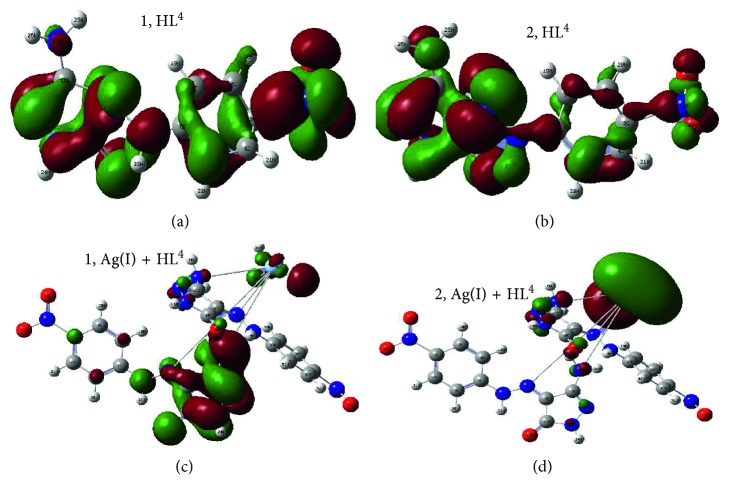
Images of frontier molecular orbital's (HOMO,1 and LUMO,2) for HL^4^ ligand and its Ag(I) complex.

The *d*-spacing upon two following layers was extracted from the Bragg equation: *nλ* = 2*d* sin(*θ*) at *n*=1. The crystallite sizes calculated for all tested compounds appeared excellently in nanometer range. This feature devotes the application towards antitumor activity as well as DNA binding study. Also, *ε* index (crystal strain) and *δ* index (dislocation density) were calculated by known relations [45] equation. The two indexes are reflecting the dislocation inside the compound network. The lower their values, the higher the quality of compounds. Also, Miller indexes (*hkl*) were calculated on the bases of cubic feature for investigated crystals. The calculated values display mainly the parallelism of planes towards *y*- and *z*-axes while, intercepts with *x*-axis, except for Ag(I)-HL^2^ and Ag(I)-HL^4^ complexes. This reflects the relatively perfect crystal building towards the axes with most compounds. SEM is another technique used to obtain a clear view about the surface topography for tested compounds (Figure 6S). The images of four organic ligands were found as needles or sticks by a regular way. Also, the high aggregation observed with HL^4^ ligand may reflect its distinguished nanometer size of separate crystals which coincide with XRD data. However, the images of complexes are completely different from that of corresponding ligands, which initially reflects the purity. Moreover, the spherical shape is the feature observed, which indicates the impact of metal central atom in the aggregation process of particles.

### 3.7. DNA Binding Studies

**Figure 7:**
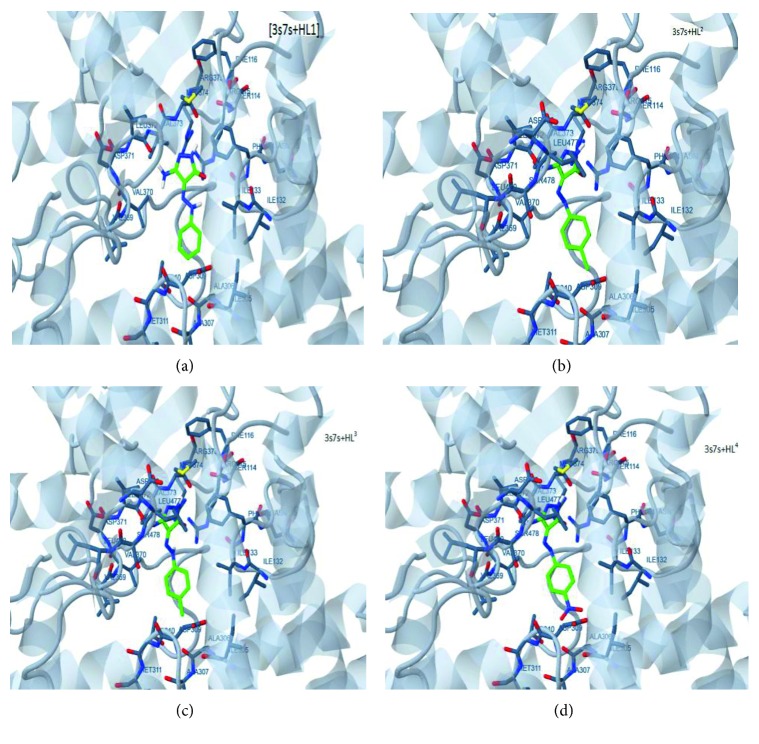
High interacting complexes for 3s7s protein with HL^1–4^ ligands.

The degree of interaction of Ag(I) complexes against CT-DNA was examined by the spectrophotometric titration method. The freshly prepared solutions (as reported in experimental part) were scanned along the applied wave length range (200–900 nm) against referenced solutions (blank). The intrinsic binding constants (*K*_b_) were calculated at *λ*_max_ for each charge transfer band; 364.7, 366.4, 365.1, and 458 nm attributing to the binding yields of silver complexes (1–4) and DNA. The absorption relationships for all tested compounds are displayed in Figure 7S. An observable bathochromic effect for new CT band is conjugated with progressive increase DNA amount [46]. The red shift observed is found in the range 1–4 nm, with all investigated compounds. This indicates the formation of new compounds yielded from the binding with DNA helix. Also, a gradual altitude in the absorption is the other effective indicator. The higher the binding constant, the higher the stability of coupled DNA helix. The coupling process is mainly proper with occluding inside major and minor grooves of DNA, whereas the coupling process attributing to silver complexes may prolonged by electrostatic attraction for cationic coordination sphere as well as occlusion inside grooves. The coupling stabilization may be facilitated by the broadness of occluded compound surface as well as a charged coordination sphere (with silver complex). The occlusion process causes reorganization inside CT-DNA, which required partial disassembly or deterioration for the double helix at the exterior phosphate leading to form cavity suitable for entering [47]. The bathochromic shift is corelated with the substituent's effect by a direct relation with electron withdrawing character (*σR*). Hammett's equation established the significant effect of *p*-substituent's on the binding constant value (*σR* and *K*_b_) [48]. The constant of binding was arranged by 1.29 × 10^4^, 1.82 × 10^4^, 3.57 × 10^3^, and 2.4 × 10^4^, attributing to complexes for HL^1–4^, respectively, in agreement with Hammett's relation. The binding constants reflect an acceptable interaction between the complexes and CT-DNA. The higher the inductive effect, the higher the interacting ability with DNA helix. Other essential plots against *σR* (Figure 4) were extracted to clarify the relationships as; Δ*E*, PK_a_, (docking parameter), and log *P*. A direct relation was shinned with Δ*E* (energy gaps) and PK_a_ plots, while a reverse with log *P*. The effective impact for log *P* value is appearing with the minimization of its value.

### 3.8. Antitumor Study

All synthesized complexes were screened against three cell lines (HEPG2, MCF-7, and HCT116) to establish a view about their antitumor activity. The obtained results propose the cytotoxic feature of complexes towards the tested cell lines in comparison with congruent ligands. The IC_50_ values are represented in Table 4 and graphed in Figure 8S. The cytotoxic indicator IC_50_ is considered a good anticancer parameter on behalf of other significant indexes such as DNA fragmentation or morphology of the cell. Based on this vision, experiments were conducted on all compounds to identify their impact on reducing cells or not through morphological index. To achieve that, cells were treated with IC_50_ for selected high impacted compounds (HL^3-4^ and their complexes). MCF-7 breast cancer was the chosen cell line. After treatment with IC_50_ of selected compounds, MCF-7 cells were incubated for 24 hours. Cells were fixed using Carnoy's fixative and stained by fluorescent stain [49]. The estimation was based on the nucleus shape, nucleus density, foci presence, and the number of cells. The outcome data clearly showed that nucleus remained in its regular shape and that there were no condensation and no degradation for detected nucleus (Figure 8S). Lack of micronucleus inside cytoplasm revealed that ligands and their Ag(I) complexes are not clastogenic agents, which may consider a very good feature. Also, cells preserved their capability to produce foci which means that the compounds may have no impact towards penetrating layers and have no ability to solve cell-to-cell connections. This may be considered useful for them because they do not help cells to spread, where they can be killed in situ (Figure 5). In terms of cell numbers, picture has assured the compatibility and regularity with the datum proposed by SRB assay. In the light of former results and discussion, ligands and complexes may be treated as in situ cytotoxic but not genototoxic.

### 3.9. Computational Utilizations

#### 3.9.1. Kinetics

The kinetics calculations are a significant study that gives clear view about the impact of metal atom occluded with organic molecules upon the thermal degradation attitude over whole compound (Table 5). *E*, ∆*H*, ∆*S*, and ∆*G* are the parameters computed over two degradation stages in each investigating compound. These parameters were calculated based on evaluating fraction decompose (*α*) at variable temperatures inside step borders. Coats and Redfern [23] and Horowitz and Metzger equations [28] are the two comparative methods used. The relationship graphs are exhibited in Figures 9AS and 9BS. ∆*H* = *E* − *RT* and ∆*G* = ∆*H*− *T*∆*S* were the equations used. Entropy of activation (∆*S*) was extracted from the equation Δ*S* ^∗^=*R*ln(*Ah*/*K*_*b*_ *T*_*s*_), where *h*, *K*_b_, and *T*_s_ are Plank's constant, Boltzmann constant, and DTG peak midpoint temperature, respectively. The following notes are grouped into calculated values: (i) The values of activation energy are suffering reduction with complexes in comparison with free ligands. This is a good evidence for the weakness appearing on the bonds of organic compound as a result of metal coordination. (ii) There is an observable increase in the activation energy from one step to another due to the reduction of decomposition rate (endothermic steps) from the original molecule. This will lead to increase of ∆*G* values in the following stages. (iii) The altitude of activation entropies (∆*S,* by negative sign) for complexes upon their corresponding ligands reflects the high-ordered complexes than the free ligands and/or slow reaction rate.

#### 3.9.2. Structural Optimization Parameters

Applying Gaussian 09 software, the best orientation for atoms inside the structures was extracted. DFT/B3LYP is a proper method for this purpose. The optimization procedure exerted essential output files (chk and log). The visualization for chk file, HOMO, and LUMO images and their energy values were obtained. The frontier, HOMO, and LUMO images belong to free ligands and their complexes are displayed in Figure 6 and Figures 10AS and 10BS. The HOMO level for pyrazolone ligands were centered on pyrazol-5(*4H*)-one, moiety which includes the atoms interested in bonding. While, the LUMO level were spread over a whole molecule. However, the attitude of HOMO and LUMO levels in silver complexes were centered on the metal atom. This is normal with such electron-dense atom as silver. All effective parameters tabulated in Table 3S were computed concerning to frontier energy gaps (*E*_LUMO_ – *E*_HOMO_, Δ*E*). The indexes *χ* (electronegativity), *μ* (chemical potential), *η* (global hardness), *S* (global softness), *ω* (electrophilicity), and *ϭ* (absolute softness) were calculated using referenced equations [50, 51]. Using electrophilicity index (*ω*), the toxicity and reactivity of tested compounds can be evaluated. This will give an acceptable expectation for the biological feature of compounds by which, the excellence for complexes upon their corresponding ligands will be expected with the progression for Ag(I)-HL^4^ complex. As well as, the constancy and electron affinity can be evaluated from two opposing indexes; *η* and *ϭ* [52, 53]. Also, the superiority was recorded with the complexes in comparison with their congruous ligands. Other two opposing indexes (*S* and *η*) attributing to the degree of softness displayed the soft character of all compounds with the priority of complexes. This indicates an expected distinct biological behavior for complexes. Furthermore, frontier energy gaps in complexes were reduced from original ligands. This is due to the effective impact of metal atom, which is favored in biological activity [54].

#### 3.9.3. Considerable Log File Parameters and QSAR

Other additive parameters were deducted from log file data, from which important data are drawn (Table 4S) that give accurate specifications for structural form and confirmed it. Significant charges of coordinating atoms (N^8^, N^15^, N^36^, and N^43^) before (free ligand) and after complexation suffer reduction overall. Moreover, the net charge of silver ion is highly reduced due to charge transfer accompanied with coordination (L → M). Two bond lengths contributing with donor atoms (C^11^–N^15^ and C^12^–N^8^) were estimated for ligands only, while they could not be technically computed for complexes. So, effective comparison was absent. Oscillator strength (*ʄ*) was extracted for all studied compounds and compared. An overall lessening was recorded with the complexes values. This is a good indicator for facilitating electronic transition inside the whole complexes molecules [55]. The dipole moment values were extracted and display variable features in comparative view. Two of them suffer enhancement in complexes and the others significantly reduced. The enhancement may reflect increase in polarity over a molecule, whilst, the reduction purpose a reverse feature. QSAR parameters (Table 6) were obtained after optimizing the structures of all tested compounds using HyperChem (8.1) program. Surface area and the volume of tested compounds showed a significant increase in complexes compared to ligands. Also, reactivity and polarizability were generally enhanced in complexes with the priority of Ag(I)-HL^4^ complex in agreement with the previously displayed. Moreover, log *P* values were estimated and displayed excellence with Ag(I)-HL^4^ complex and its ligand. Partition coefficient (log *P*) points to the biological feature of tested compound by a reverse relation.

#### 3.9.4. Docking Interpretation

In the last few decades, a great revolution is seen in drug design industry, which extended to preemptive theoretical studies to show the expected biological efficiency of proposed drug. Autodock 4.2. tools software is one of these tools used for this purpose. The program designed for simulation about the interaction between proposed drug (inhibitor) and protein receptors attributes to infected cells. 3s7s, 3e1r, 4dk7, and 1bpb were the receptors used for breast, colon, and liver cancers besides CT-DNA polymerase. The docking feature of all organic compounds was studied to evaluate the inhibition activity towards the selected proteins. The output internal energies computed over PDB files are tabulated (Table 5S). p*K*_a_ (dissociation constant) value was calculated for each compound, which is considered a biopharmaceutical measure for drug-likeness feature. This constant is reflecting the degree of ionization for compound. The high p*K*_a_ value means high degree of polarity around hydrogen atoms of function groups. This will lead to increase in the number of H-bonding between the proposed drug (ligand) and the protein receptor. Also, a direct relation between p*K*_a_ value and *σR* (Figure 4) goes parallel with the former aspect. Computed different energies showed distinct correlations for docked drugs (ligands) with breast cancer followed by DNA polymerase proteins [56]. The best docking complexes (Figure 7),(3s7s-HL^1–4^) display high occlusion for drugs (ligands) which centered inside protein helix. Also, the binding towards DNA (1bpb) is highly satisfactory for the inhibition feature while the other docking complexes with 3e1r and 4dk7 proteins display binding in moderate level (Figure 11S). 2D plots (Figure 12S) verify the intense H-bonding with 3s7s protein in comparison with others. This study shows the promising inhibition activity towards breast cancer as well as CT-DNA which strongly and strangely agreeable with practical data that reported in our study.

## 4. Conclusion

A series of tetrahedral Ag(I)-pyrazolone complexes were synthesized and characterized. All compounds investigated were found in the nanometer range based on UV-Vis and XRD spectra. CT-DNA study yields intrinsic binding constants by the arrangement attributing to Hammett's postulation. Also, antitumor activity offered a promising feature for the Ag(I)-HL^4^ complex. Structural formulae of all synthesized compounds were optimized theoretically. Also, simulation for the interaction between tested drugs and types from infected cell proteins was achieved.

## Figures and Tables

**Table 1:** Analytical and physical data for Ag(I)-pyrazolone (HL^1–4^) complexes.

Compounds (empirical formula, calcd./found)	Λ_m_ (Ω^−1^·cm^2^·mol^−1^)	Color	Elemental analysis (%) calcd. (found)			
			C	H	N	M
(1) HL^1^(C_9_H_9_N_5_O) (203.20)	—	Red	53.20 (53.18)	4.46 (4.46)	34.47 (34.44)	—
(2) [Ag(HL^1^)_2_](NO_3_) (576.27)	57.31	Brown	37.52 (37.51)	3.15 (3.16)	26.74 (26.72)	18.72 (18.72)
(3) HL^2^(C_9_H_8_N_5_OCl) 237.64/239	—	Red	45.49 (45.50)	3.39 (3.41)	29.47 (29.45)	—
(4) [Ag(HL^2^)_2_](NO_3_) 645.15/647.66	55.21	Reddish brown	33.51 (33.52)	2.50 (2.50)	23.88 (23.89)	6.72 (16.71)
(5) HL^3^(C_10_H_11_N_5_O) 217.23/218	—	Faint brown	55.29 (55.31)	5.10 (5.11)	32.24 (32.25)	—
(6) [Ag(HL^3^)_2_](NO_3_) 2H_2_O 640.36/605	51.06	Brown	37.51 (37.50)	4.09 (4.11)	24.06 (24.10)	6.84 (16.86)
(7) HL^4^(C_9_H_8_N_6_O_3_) (248.20)	—	Faint brown	43.55 (43.55)	32.49 (32.47)	33.86 (33.85)	—
(8) [Ag(HL^4^)_2_](NO_3_) 666.27/665.22	56.12	Deep brown	32.45 (32.43)	2.42 (2.41)	27.33 (27.35)	6.19 (16.20)

**Table 2:** DMSO-*d*_6_, ^1^H NMR, and ^13^C NMR data (ppm) for Ag(I)-pyrazolone complexes.

Compound	*δ*(s, NH_2_)	*δ*(m, Ar-CHs, amidic-NH); (s, CH_3_)	*δ*(s, NH_h_)	*δ*(CH_3_)	*δ*(2C, Ar), (2C, Ar)	*δ*(C, Ar), (*p*-C, Ar)	*δ*(C=O)	*δ*2(C=N)
Ag(I) + HL^1^	5.86	7.13–7.52	10.55	—	115.48, 128.89	141.32, 118.35	161.21	150.47
Ag(I) + HL^2^	5.90	7.43–7.60	10.56	—	117.39, 129.69	141.34, 125.61	161.30	150.37
Ag(I) + HL^3^	5.86	7.21–7.44; 2.3	10.54	20.92	115.60, 130.33	139.90, 129.30	161.89	153.81
Ag(I) + HL^4^	6.09	7.78–8.10	10.69	—	117.36, 128.38	148.33, 143.09	158.04	150.25

**Table 3:** XRD computed data for Ag(I)-pyrazolone (HL^1–4^) complexes.

Compounds	Size (Å)	2*θ*	Intensity	*d*-spacing (Å)	*ε*	*δ*(Å^−2^)	FWHM	1000 sin^2^ *θ*	*a* ^2^	*hkl*
(1) HL^1^	6.872	12.96	1572	6.8255	0.1191	0.0214	0.2121	12.737	46.586	100
(2) [Ag(HL^1^)_2_](NO_3_)	5.585	10.92	222	8.0956	0.1737	0.0153	0.2605	9.054	65.536	100
(3) HL^2^	6.460	14.60	298	6.0623	0.1470	0.0272	0.2216	16.145	36.752	100
(4) [Ag(HL^2^)_2_](NO_3_)	5.880	26.68	384	3.3385	0.0682	0.0897	0.2531	53.236	11.146	331
(5) HL^3^	2.842	14.12	570	6.2673	0.2650	0.0255	0.5134	15.107	39.277	100
(6) [Ag(HL^3^)_2_](NO_3_)2H_2_O	2.561	38.02	254	2.3648	0.1105	0.1788	0.5982	106.102	5.593	100
(7) HL^4^	1.980	27.65	2086	3.2236	0.1954	0.0962	0.7532	57.101	10.391	100
(8) [Ag(HL^4^)_2_](NO_3_)	4.604	28.08	172	3.1752	0.0829	0.0992	0.3242	58.85	10.083	311

**Table 4:** Cytotoxic activity of tested complexes against human cancer cell lines.

Cell type	IC_50_ (*µ*g/ml)								
	HL^1^	Ag(I)-HL^1^	HL^2^	Ag(I)-HL^2^	HL^3^	Ag(I)-HL^3^	HL^4^	Ag(I)-HL^4^	Doxorubicin
MCF-7	60.4	60.8	57.7	78.8	14.5	12.81	1.976	16.66	0.168
HepG2	71.3	62.32	63.52	88.5	17.8	11.88	15.86	12.52	0.777
HCT116	36.5	26.8.5	52.6	45.8	20.4	12.8	14.8	8.42	0.9255

**Table 5:** Kinetic parameters by using Coats–Redfern (CR) and Horowitz–Metzger (HM) methods.

Comp.	Step	Method	Kinetic parameters					
			*E* (J·mol^−1^)	*A* (S^−1^)	Δ*S* (J·mol^−1^·K^−1^)	Δ*H* (J·mol^−1^)	Δ*G* (Jmol^−1^)	*r*
(1) HL^1^	1st	CR	5.67*E* + 04	9.77*E* + 03	−1.72*E* + 02	5.27*E* + 04	1.34*E* + 05	0.99993
		HM	6.43*E* + 04	1.07*E* + 05	−1.52*E* + 02	6.04*E* + 04	1.33*E* + 05	
	2nd	CR	3.58*E* + 04	1.30*E* + 06	−1.35*E* + 02	3.02*E* + 04	1.22*E* + 05	
		HM	4.55*E* + 04	9.47*E* + 00	−2.33*E* + 02	3.99*E* + 04	1.98*E* + 05	

(2) [Ag(HL^1^)_2_](NO_3_)	1st	CR	4.49*E* + 04	9.46*E* + 03	−1.73*E* + 02	4.09*E* + 04	1.26*E* + 05	0.99993
		HM	5.25*E* + 04	2.48*E* + 03	−1.84*E* + 02	4.84*E* + 04	1.39*E* + 05	
	2nd	CR	3.43*E* + 04	2.20*E* + 06	−1.31*E* + 02	2.85*E* + 04	1.20*E* + 05	
		HM	4.61*E* + 04	7.76*E* + 00	−2.35*E* + 02	4.03*E* + 04	2.05*E* + 05	

(3) HL^2^	1st	CR	3.62*E* + 04	4.94*E* + 04	−1.59*E* + 02	3.21*E* + 04	1.11*E* + 05	0.99993
		HM	4.42*E* + 04	2.28*E* + 02	−2.04*E* + 02	4.00*E* + 04	1.42*E* + 05	
	2nd	CR	4.16*E* + 04	1.09*E* + 06	−1.37*E* + 02	3.54*E* + 04	1.37*E* + 05	
		HM	5.55*E* + 04	2.57*E* + 01	−2.25*E* + 02	4.94*E* + 04	2.16*E* + 05	

(4) [Ag(HL^2^)_2_](NO_3_)	1st	CR	4.32*E* + 04	2.34*E* + 04	−1.66*E* + 02	3.88*E* + 04	1.27*E* + 05	
		HM	5.14*E* + 04	6.58*E* + 02	−1.96*E* + 02	4.70*E* + 04	1.51*E* + 05	
	2nd	CR	5.85*E* + 04	1.04*E* + 05	−1.57*E* + 02	5.22*E* + 04	1.70*E* + 05	
		HM	7.38*E* + 04	5.11*E* + 02	−2.01*E* + 02	6.75*E* + 04	2.19*E* + 05	

(5) HL^3^	1st	CR	3.45*E* + 04	6.69*E* + 04	−1.57*E* + 02	3.04*E* + 04	1.09*E* + 05	0.99993
		HM	4.46*E* + 04	2.29*E* + 02	−2.04*E* + 02	4.04*E* + 04	1.43*E* + 05	
	2nd	CR	2.18*E* + 04	1.79*E* + 07	−1.14*E* + 02	1.56*E* + 04	9.97*E* + 04	
		HM	3.37*E* + 04	4.47*E* − 01	−2.59*E* + 02	2.76*E* + 04	2.19*E* + 05	

(6) [Ag(HL^3^)_2_](NO_3_)2H_2_O	1st	CR	4.73*E* + 04	2.24*E* + 04	−1.64*E* + 02	4.41*E* + 04	1.07*E* + 05	0.99993
		HM	5.47*E* + 04	2.76*E* + 05	−1.43*E* + 02	5.15*E* + 04	1.07*E* + 05	
	2nd	CR	4.30*E* + 04	2.08*E* + 04	−1.67*E* + 02	3.88*E* + 04	1.24*E* + 05	
		HM	5.16*E* + 04	1.05*E* + 03	−1.92*E* + 02	4.74*E* + 04	1.46*E* + 05	

(7) HL^4^	1st	CR	3.97*E* + 04	2.89*E* + 04	−1.64*E* + 02	3.56*E* + 04	1.17*E* + 05	0.99993
		HM	4.79*E* + 04	6.18*E* + 02	−1.96*E* + 02	4.37*E* + 04	1.41*E* + 05	
	2nd	CR	3.72*E* + 04	1.94*E* + 06	−1.32*E* + 02	3.10*E* + 04	1.29*E* + 05	
		HM	4.98*E* + 04	8.93*E* + 00	−2.34*E* + 02	4.36*E* + 04	2.17*E* + 05	

(8) [Ag(HL^4^)_2_](NO_3_)	1st	CR	3.59*E* + 04	1.41*E* + 05	−1.51*E* + 02	3.15*E* + 04	1.12*E* + 05	0.99993
		HM	4.46*E* + 04	1.15*E* + 02	−2.10*E* + 02	4.02*E* + 04	1.52*E* + 05	
	2nd	CR	5.76*E* + 04	1.22*E* + 05	−1.55*E* + 02	5.14*E* + 04	1.67*E* + 05	
		HM	6.96*E* + 04	2.96*E* + 02	−2.05*E* + 02	6.35*E* + 04	2.16*E* + 05	

**Table 6:** QSAR computation for pyrazolone ligands and their Ag(I) complexes.

Function	HL^1^	Ag(I)-HL^1^	HL^2^	Ag(I)-HL^2^	HL^3^	Ag(I)-HL^3^	HL^4^	Ag(I)-HL^4^
Surface area (approx) (Å^2^)	303.45	570.44	338.10	648.39	345.78	685.84	351.56	677.32
Surface area (grid) (Å^2^)	396.20	703.28	421.12	755.88	424.55	773.68	427.04	648.34
Volume (Å^3^)	611.36	1234.02	655.15	1322.62	664.82	1364.65	728.68	1410.40
Hydration energy (kcal/mol)	16.61	−20.40	−16.23	−20.24	−15.36	−18.13	−29.03	−41.97
Log *P*	0.36	−0.89	0.14	−1.33	0.52	−0.58	−3.48	−8.57
Reactivity (Å^3^)	58.85	114.45	63.57	123.89	63.13	123.02	64.10	124.95
Polarizability (Å^3^)	21.17	42.00	23.10	45.86	23.01	45.67	23.08	45.82

## Data Availability

The data used to support the findings of this study are available from the corresponding author upon request.

## Supplementary Materials

Supplementary Materials Table 1S: characteristic IR spectral bands (cm^−1^) for Ag(I)-pyrazolone (HL^1–4^) complexes. Table 2S: plausible TGA data for Ag(I)-pyrazolone (HL^1–4^) complexes. Table 3S: energy parameters (eV) applying DFT method. Table 4S: significant log file data for pyrazolone ligands and their Ag(I) complexes. Table 5S: energy values (kcal/mol) for pyrazolone-protein docked complexes. Figure 1S: ^1^H NMR spectra of pyrazolone derivatives, HL^(1–4)^. Figure 2S: mass spectra of two elected ligands. Figure 3S: ^1^H NMR spectra of three Ag(I)-pyrazolone derivatives. Figure 4S: mass spectra of selected ligands and two Ag(I) complexes. Figure 5S: X-ray patterns of three pyrazolone derivatives and their Ag(I) complexes. Figure 6S: SEM images of pyrazolone derivatives, HL^(1–4)^ and their Ag(I) complexes. Figure 7S: spectrophotometric plots for DNA binding towards Ag(I) complexes (1–4). Figure 8S: dose response curves of HL (1–4) ligands and their Ag(I) complexes against MCF-7, HEPG-2 and HCT-116 cancer cells, cells were treated with various concentrations of ligands and complexes incubated for 72 hrs. %5 CO_2_. Figure 9AS: kinetic plots using Coats–Redfern and Horowitz–Metzger for pyrazolone ligands, HL (1–4). Figure 9BS: kinetic plots using Coats–Redfern and Horowitz–Metzger for pyrazolone-Ag(I) complexes. Figure 10AS: images of frontier molecular orbital's (HOMO,1 and LUMO,2) for pyrazolone ligands (HL^1–3^). Figure 10BS: images of frontier molecular orbital's (HOMO,1 and LUMO,2) for Ag(I)-HL^1–3^ complexes (A–C, respectively). Figure 11S: interacting complexes appeared with 3e1r, 4dk7, and 1bpb proteins and HL^1–4^ ligands. Figure 12S: 2D plots for interacting complexes appeared with 3e1r, 4dk7, 1bpb, and 3s7s proteins proteins and HL^1–4^ ligands.
