# Supplementary material for: Synthesis of Pyrazolone Derivatives and Their Nanometer Ag(I) Complexes and Physicochemical, DNA Binding, Antitumor, and Theoretical Implementations
Source: Bioinorg Chem Appl. 2018 May 14;2018:2727619. doi: 10.1155/2018/2727619 (PMC5976992; doi:10.1155/2018/2727619)
Supplement: Supplementary Materials — Table 1S: characteristic IR spectral bands (cm−1) for Ag(I)-pyrazolone (HL1–4) complexes. Table 2S: plausible TGA data for Ag(I)-pyrazolone (HL1–4) complexes. Table 3S: energy parameters (eV) applying DFT method. Table 4S: significant log file data for pyrazolone ligands and their Ag(I) complexes. Table 5S: energy values (kcal/mol) for pyrazolone-protein docked complexes. Figure 1S: 1H NMR spectra of pyrazolone derivatives, HL(1–4). Figure 2S: mass spectra of two elected ligands. Figure 3S: 1H NMR spectra of three Ag(I)-pyrazolone derivatives. Figure 4S: mass spectra of selected ligands and two Ag(I) complexes. Figure 5S: X-ray patterns of three pyrazolone derivatives and their Ag(I) complexes. Figure 6S: SEM images of pyrazolone derivatives, HL(1–4) and their Ag(I) complexes. Figure 7S: spectrophotometric plots for DNA binding towards Ag(I) complexes (1–4). Figure 8S: dose response curves of HL (1–4) ligands and their Ag(I) complexes against MCF-7, HEPG-2 and HCT-116 cancer cells, cells were treated with various concentrations of ligands and complexes incubated for 72 hrs. %5 CO2. Figure 9AS: kinetic plots using Coats–Redfern and Horowitz–Metzger for pyrazolone ligands, HL (1–4). Figure 9BS: kinetic plots using Coats–Redfern and Horowitz–Metzger for pyrazolone-Ag(I) complexes. Figure 10AS: images of frontier molecular orbital's (HOMO,1 and LUMO,2) for pyrazolone ligands (HL1–3). Figure 10BS: images of frontier molecular orbital's (HOMO,1 and LUMO,2) for Ag(I)-HL1–3 complexes (A–C, respectively). Figure 11S: interacting complexes appeared with 3e1r, 4dk7, and 1bpb proteins and HL1–4 ligands. Figure 12S: 2D plots for interacting complexes appeared with 3e1r, 4dk7, 1bpb, and 3s7s proteins proteins and HL1–4 ligands. [file 2727619.f1.pdf]

TABLE 1S: Characteristic IR spectral bands (cm^{-1}) for Ag(I)- pyrazolone (HL^{1-4}) complexes

Compounds	ν_{asNH2}, ν_{sNH2}, ν_{OH}	ν_{NHs}	$\nu_{C=O}$	$\nu_{(C=N)s}$	δ_{NH2}	δ_{NHs}	$\nu(NO_3)$(ion)	ν_{M-N}
1) HL^1	3410,3333	3184, 3160	1671	1586, 1565	1625	1493, 1442	-------	----
2) $[Ag(HL^1)_2](NO_3)$	3319, 3247	3191	1671	1570, 1566	1595	1494,1420	1380	530
3) HL^2	3420, 3330	3195, 3180	1663	1599	1644	1522, 1488	-------	----
4) $[Ag(HL^2)_2](NO_3)$	3400, 3327	3205, 3192	1683	1591, 1583	1630	1487,	1381	560
5) HL^3	3380, 3335	3190, 3177	1667	1559,1530	1633	1505, 1464	-------	----
6) $[Ag(HL^3)_2](NO_3)2H_2O$	3410 (B.C),	3181 (B.C)	1668	1559, 1504	1629	1505, 1440	1384	511
7) HL^4	3456, 3356	3219, 3190	1672	1589, 1512	1637	1480, 1470	-------	----
8) $[Ag(HL^4)_2](NO_3)$	3445, 3338	3197(B)	1685	1573, 1512	1612	1470	1384	509

B.C, Broad band centered

TABLE 2S: Plausible TGA data for Ag(I)- pyrazolone (HL^{1-4}) complexes

Compound	Steps	Temp. range(°C)	Decomposed	Weight loss; Calcd (Found %)
1) HL^1	1st	150.2 - 250.5	$- NH_3+CO$	22.17(22.16)
	2nd	251.1 - 560.2	$- 2NH_3+2CN$	42.37(42.37)
	Residue		6C	35.46(35.47)
2) $[Ag(HL^1)_2](NO_3)$	1st	155.3 - 280.4	$-NO_2+H_2CO_3+0.5H_2$	18.92(19.01)
	2nd	282.3- 571.8	$-C_6H_6+3NH_3$	22.42(22.38)
	3rd	572.0 - 770.2	-4CN	18.06(18.06)
	Residue		$7C + AgN_3$	40.60(40.55)

3) HL^2	1st	140.1 - 310.3	$- NH_3+CO+HCl$	34.29(34.41)
	2nd	311.2 - 620.4	$- N_2H_4+2CN$	35.38(35.30)
	Residue		6C	30.33(30.29)
4) $[Ag(HL^2)_2](NO_3)$	1st	162.1 - 350.1	$-NO_2+C_2O_3+Cl_2$	29.28(29.28)
	2nd	350.2- 610.1	$-C_{12}H_{10}+NH_2+N_2$	30.73(30.76)
	3rd	610.3 - 810.8	-4HCN	16.76(16.76)
	Residue		AgN_3	23.23(23.20)
5) HL^3	1st	157.6 - 301.2	$- NH_3+CO+N_2$	33.63(33.62)
	2nd	301.2 - 631.7	$- N_2H_4+CH_4$	22.14(22.21)
	Residue		8C	44.23(44.17)
6) $[Ag(HL^3)_2](NO_3)2H_2O$	1st	67.8- 158.9	$-2H_2O$	5.63(5.62)
	2nd	160.1 - 320.9	$-NO_2+2CH_4+CO_2+CO$	23.44(23.45)
	3rd	322.1- 584.5	$-C_{12}H_{10}$	24.08(24.21)
	4th	584.7 - 805.4	$-4HCN+3N_2$	30.01(29.89)
	Residue		Ag	16.84(16.83)
7) HL^4	1st	147.6 - 301.2	$- NH_3+CO+N_2$	32.26(32.24)
	2nd	301.2 - 631.7	$- N_2H_4+CH_4$	33.87(33.90)
	Residue		8C	33.87(33.86)
8) $[Ag(HL^4)_2](NO_3)$	1st	166.1 - 350.5	$-2NO_2+ 4HCN+N_2$	34.24(34.23)
	2nd	350.5- 590.2	$-N_2O_5$	16.21(16.18)
	3rd	590.3 - 793.8	$-2C_6H_6$	23.45(23.45)
	Residue		$2C + AgN_3$	26.10(26.14)

Table 3S. Energy parameters (eV) applying DFT method

Compound	E_H (eV)	EL (eV)	(EH - EL) (eV)	El-Eh	*x*(eV)	μ(eV)	η(eV)	S(eV-1)	ω(eV)	σ(eV)
H_2L^1	-0.18521	-0.06966	-0.1156	0.11555	0.127435	-0.12744	0.057775	0.028888	0.140542	17.30852445
Ag(I)-HL^1	-0.21018	-0.19055	-0.0196	0.01963	0.200365	-0.20037	0.009815	0.004908	2.045142	101.8848701
H_2L^2	-0.19022	-0.07564	-0.1146	0.11458	0.13293	-0.13293	0.05729	0.028645	0.154219	17.45505324
Ag(I)-HL^2	-0.21571	-0.19595	-0.0198	0.01976	0.20583	-0.20583	0.00988	0.00494	2.144028	101.2145749
H_2L^3	-0.18337	-0.06722	-0.1162	0.11615	0.125295	-0.1253	0.058075	0.029038	0.13516	17.21911322
Ag(I)-HL^3	-0.19798	-0.18199	-0.016	0.01599	0.189985	-0.18999	0.007995	0.003997	2.257305	125.0781739
H_2L^4	-0.18143	-0.06156	-0.1199	0.11987	0.121495	-0.1215	0.059935	0.029968	0.123142	16.6847418
Ag(I)-HL^4	-0.21877	-0.20101	-0.0178	0.01776	0.20989	-0.20989	0.00888	0.00444	2.480507	112.6126126

TABLE 4S: Significant log file data for pyrazolone ligands and their Ag(I) complexes

Compounds	N^8	N^{15}	N^{36}	N^{43}	M	C^{11}-N^{15}	C^{12}-N^8	$D_{(Debye)}$	ʆ	E(nm)
HL^1	-0.372663	-0.810500	----	----	----	1.370778	1.290060	2.4597	0.0305	481.54
Ag(I)- HL^1	-0.379435	-0.111566	-0.358308	-0.074319	-0.170741	----	----	4.5428	0.0259	2576.38
HL^2	-0.372993	-0.170822	----	----	----	1.370611	1.290075	1.7522	0.0307	491.48
Ag(I)- HL^2	-0.382616	-0.111681	-0.361115	-0.072323	-0.164420	----	----	4.5286	0.0241	2646.8
HL^3	-0.373119	-0.174258	----	----	----	1.370975	1.289734	2.8477	0.0322	477.42
Ag(I)- HL^3	-0.361510	-0.131663	-0.355586	-0.092131	-0.259004	----	----	2.1739	0.0025	9548.13
HL^4	-0.060119	-0.064912	----	----	----	1.417723	1.334193	5.5760	0.0001	5479.51
Ag(I)- HL^4	-0.362551	-0.143065	-0.207957	0.214036	-0.189765	----	----	1.7655	0.0048	7279.67

TABLE 5S: Energy values (k cal/mol) for pyrazolone - protein docked complexes

ligands	pKa, pKb	Receptor	Est. free energy of binding	Est. inhibition constant (K_i)(uM)	vdW+ bond+ desolve energy	Electrostatic Energy	Total intercooled Energy	Frequency	Interact surface
		3s7s	-6.76	11.06	-7.91	-0.04	-7.96	80%	423.372
	1.58	3e1r	-4.75	330.07	-5.21	-0.05	-5.26	40%	416.009
HL^1	-0.81	4dk7	-3.88	1.42	-4.52	-0.05	-4.57	20%	479.222
		1bpb	-5.39	112.83	-5.88	-0.02	-5.91	50%	466.75
		3s7s	-7.89	1.64	-8.74	-0.11	-8.85	50%	455.857
	2.10	3e1r	-5.29	132.93	-5.75	-0.05	-5.81	30%	435.984
HL^2	-0.84	4dk7	-4.01	1.14	-4.53	-0.07	-4.59	10%	506.796
		1bpb	-5.89	47.95	-6.75	-0.15	-6.89	20%	515.286
		3s7s	-7.04	6.89	-7.86	-0.10	-7.96	90%	456.741
	1.05	3e1r	-4.97	225.74	-5.46	-0.02	-5.48	50%	440.961
HL^3	-0.80	4dk7	-4.14	917.05	-4.89	-0.10	-4.99	10%	517.815
		1bpb	-5.42	107.22	-6.23	-0.17	-6.40	50%	501.982
		3s7s	-8.27	863.48	-9.44	-0.11	-9.55	80%	461.009
	2.54	3e1r	-5.18	159.40	-5.92	-0.04	-5.95	30%	449.648
HL^4	-0.85	4dk7	-4.58	437.03	-5.51	+0.02	-5.49	20%	511.913
		1bpb	-6.21	28.20	-7.37	-0.17	-7.54	40%	507.894

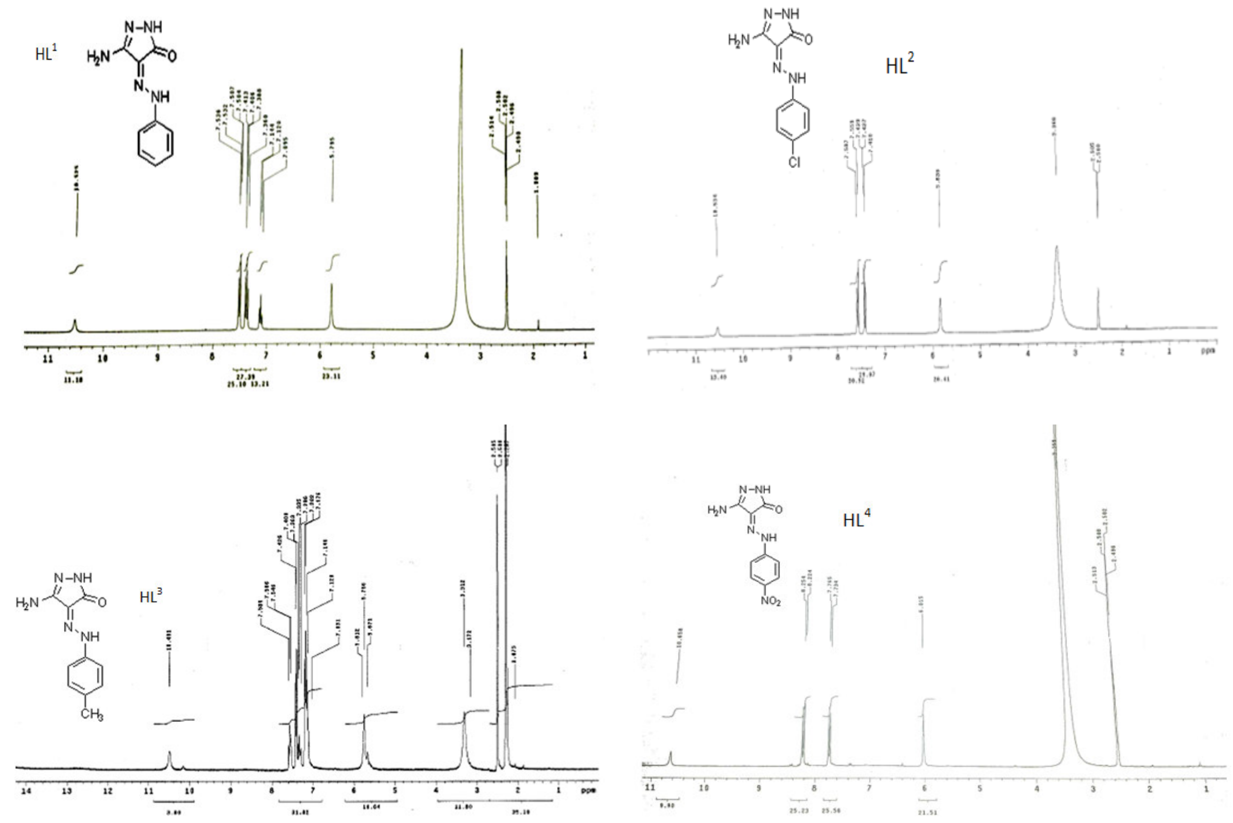

Figure 1S . ^{1}HNMR spectra of pyrazolone derivatives ,HL $^{(1-4)}$

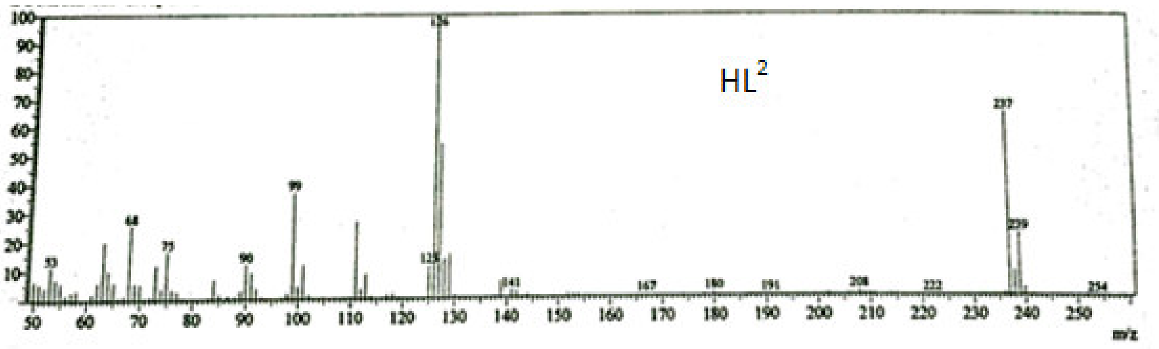

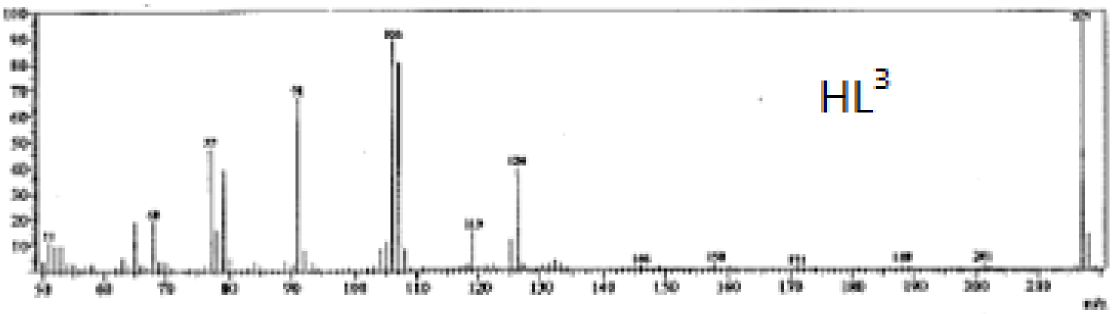

Figure 2S . Mass spectra of two elected ligands

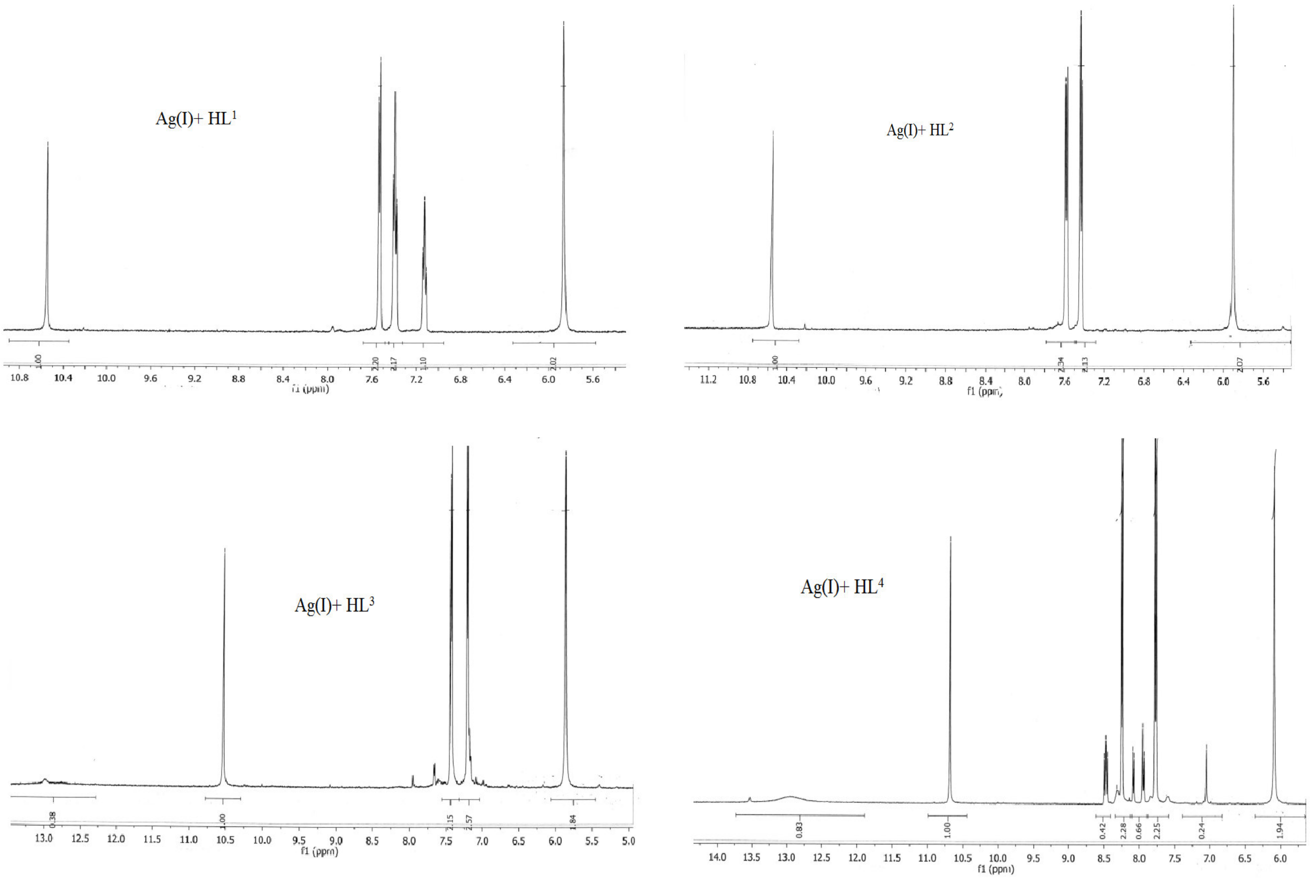

Figure 3S . ^{1}H NMR spectra of three Ag(I)- pyrazolone derivatives

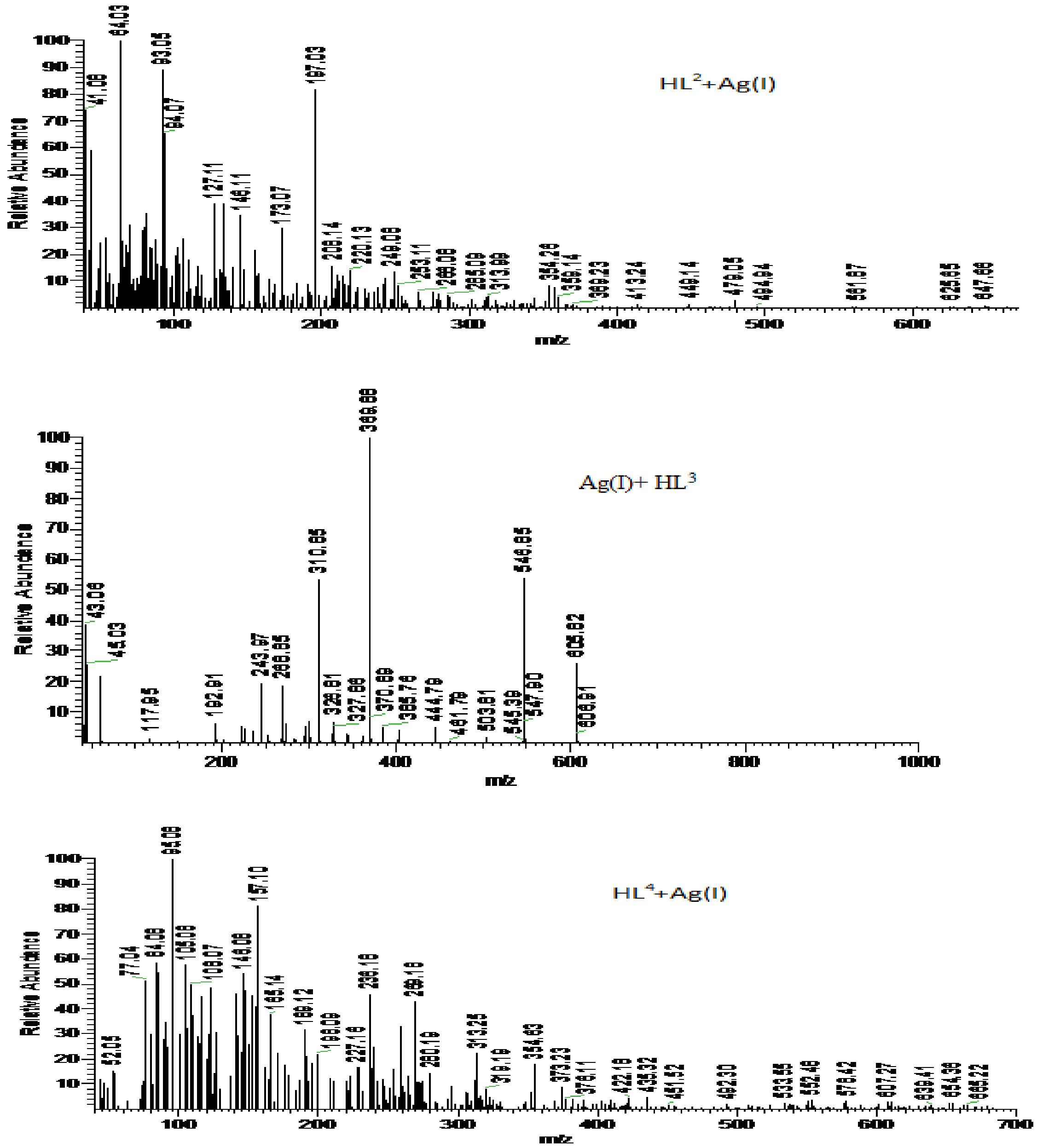

Figure 4S . Mass spectra of selected ligands and three Ag(I) complexes

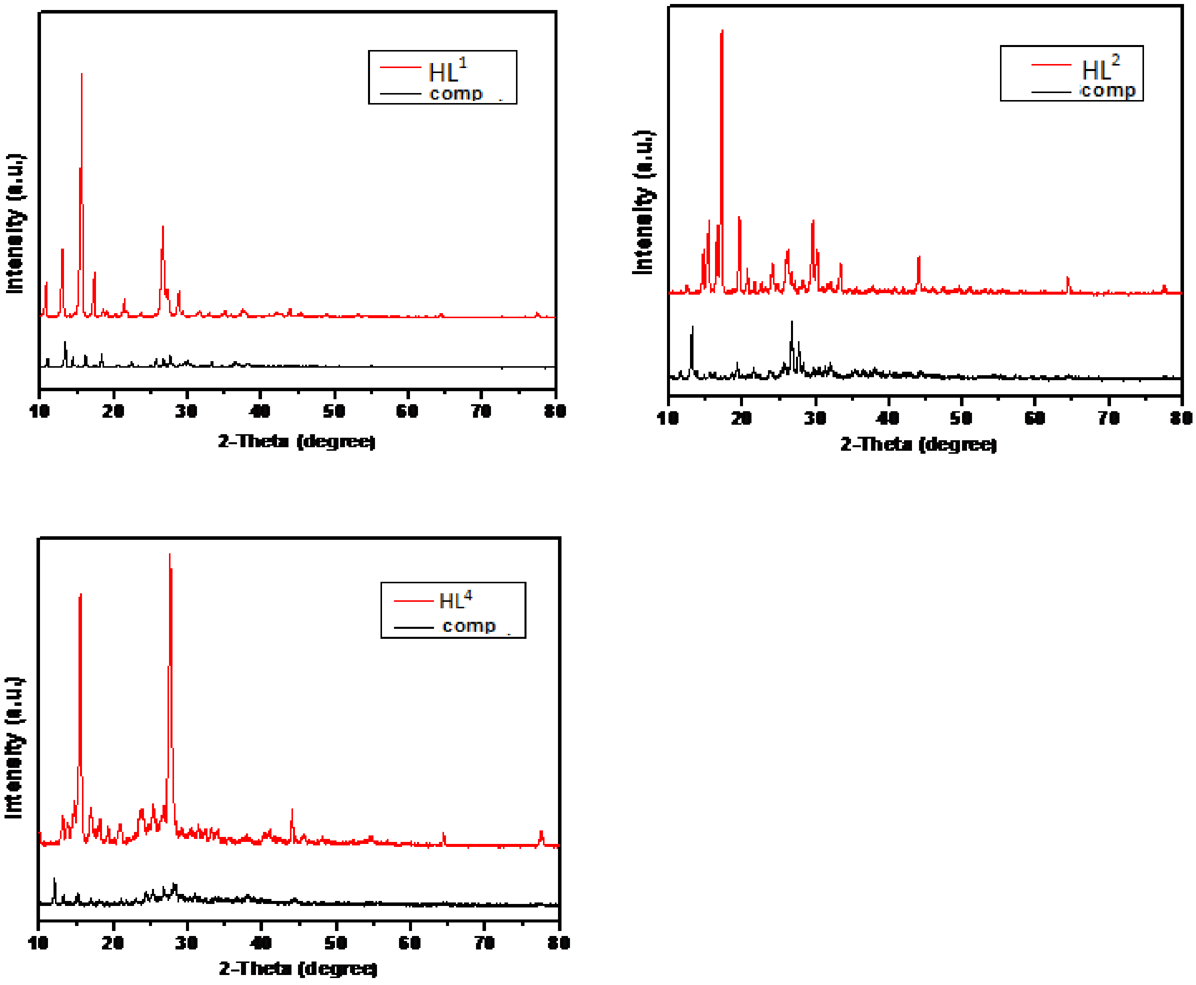

Figure 5 S. X-Ray patterns of three pyrazolone derivatives and their Ag(I) complexes

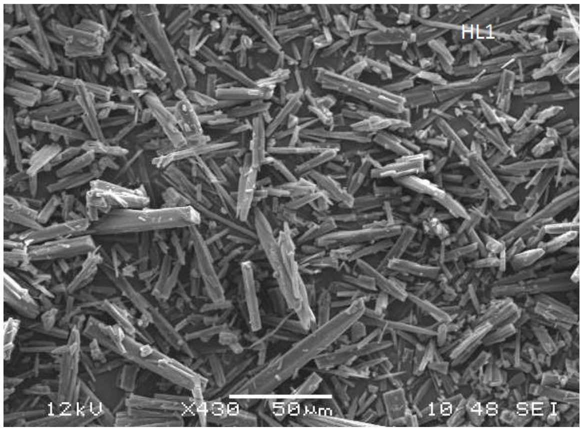

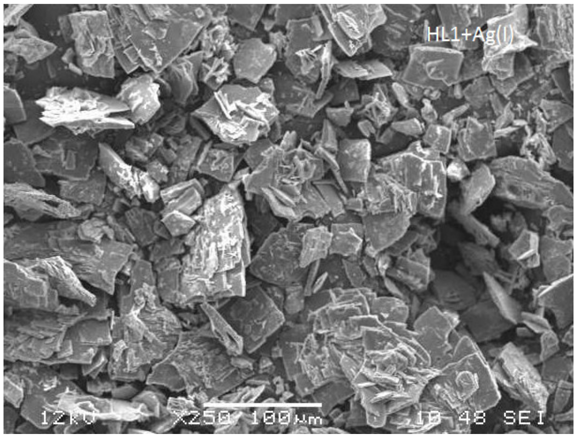

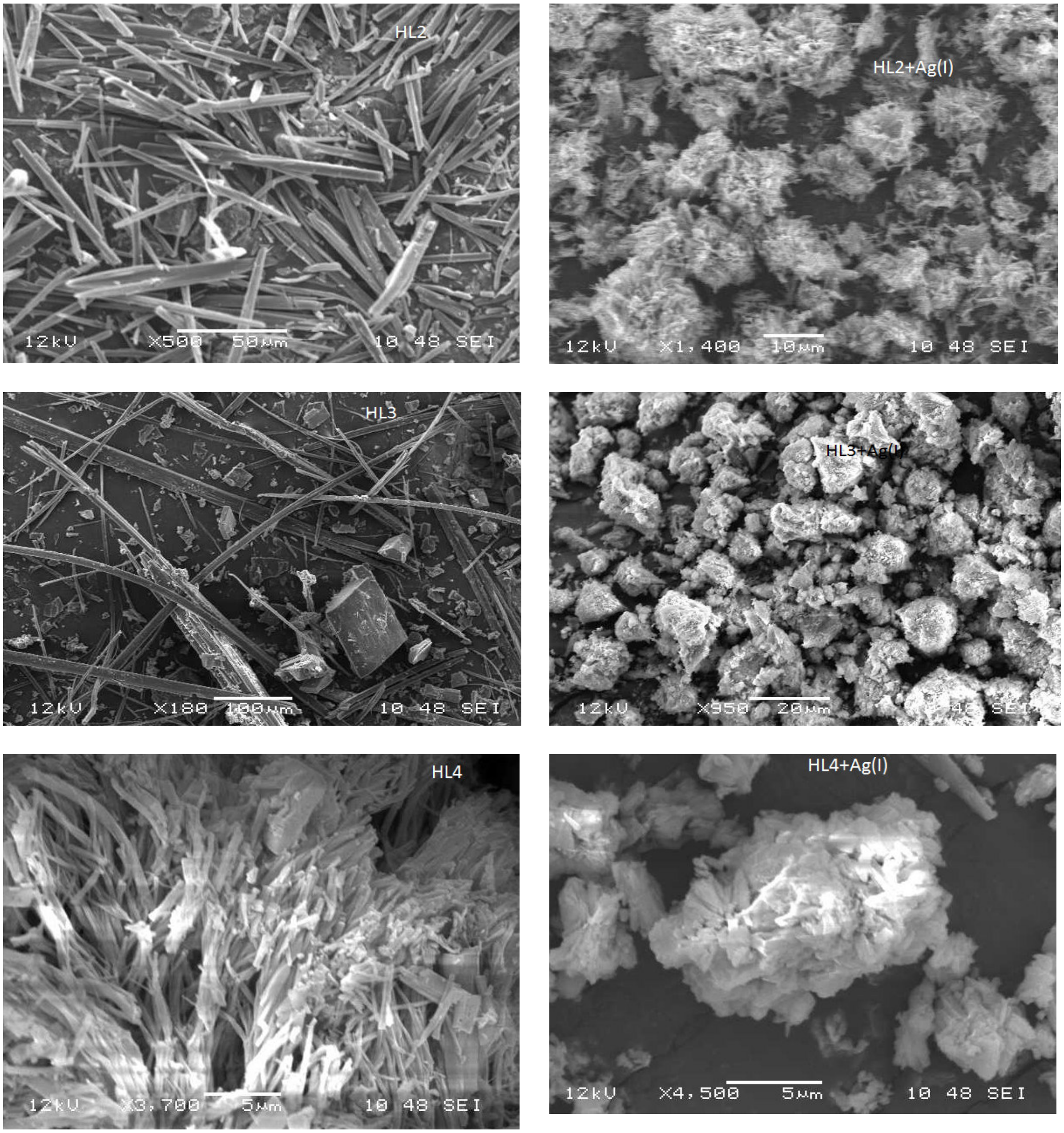

Figure 6S. SEM images of pyrazolone derivatives ,$HL^{(1-4)}$ and their Ag(I) complexes

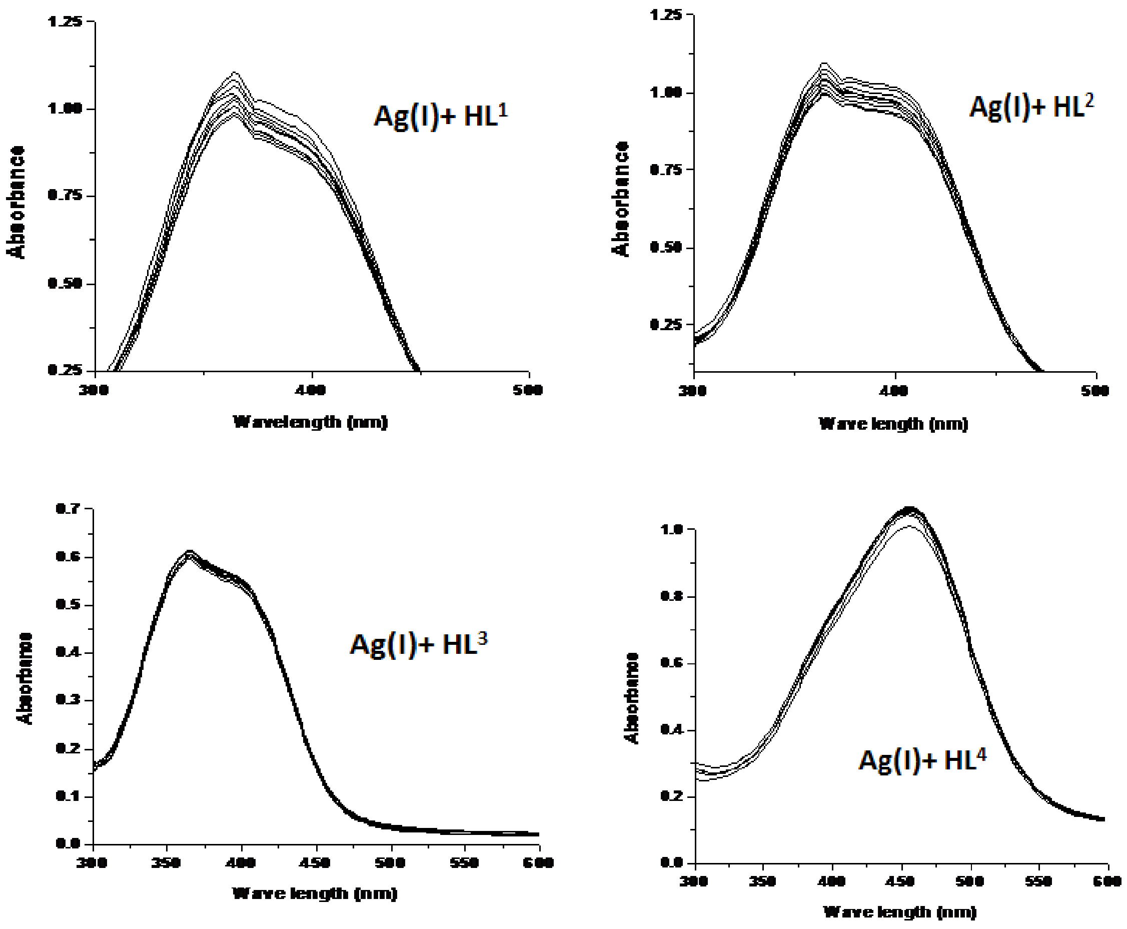

Figure 7S. Spectrophotmetric plots for DNA binding towards Ag(I) complexes(1-4)

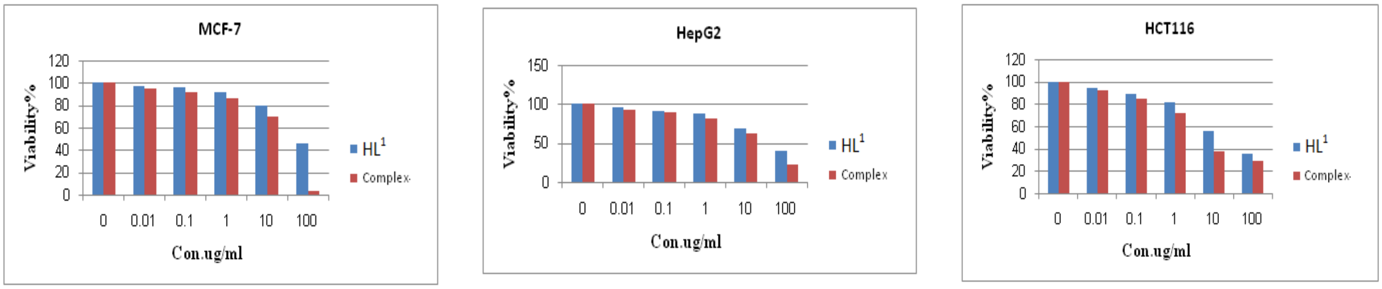

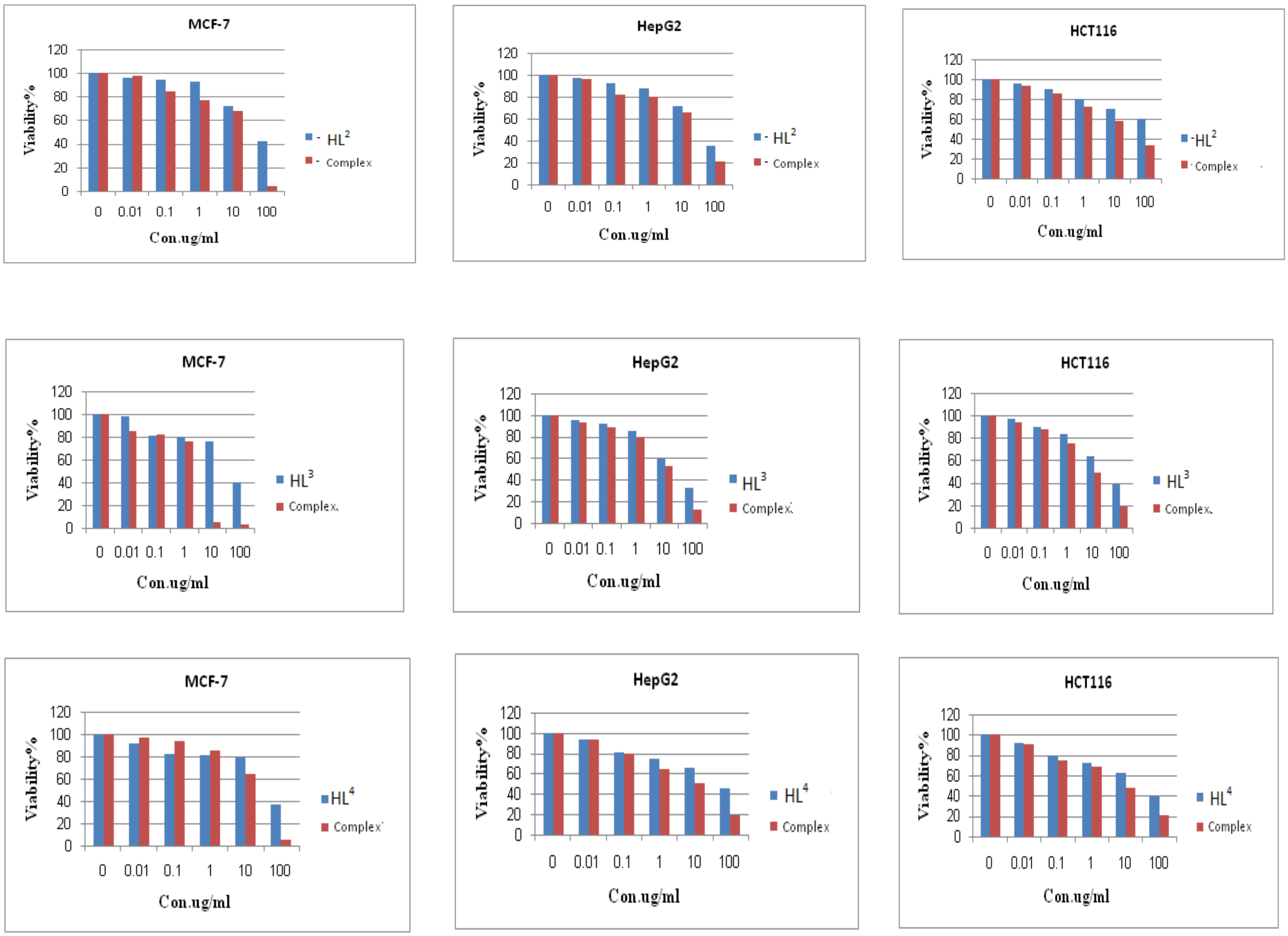

Figure 8S . Dose response curves of HL(1-4) ligands and their Ag(I) complexes against MCF-7, HEPG-2 and HCT-116 cancer cells, cells were treated with various concentrations of ligands and complexes incubated for 72 hrs. %5 CO_2.

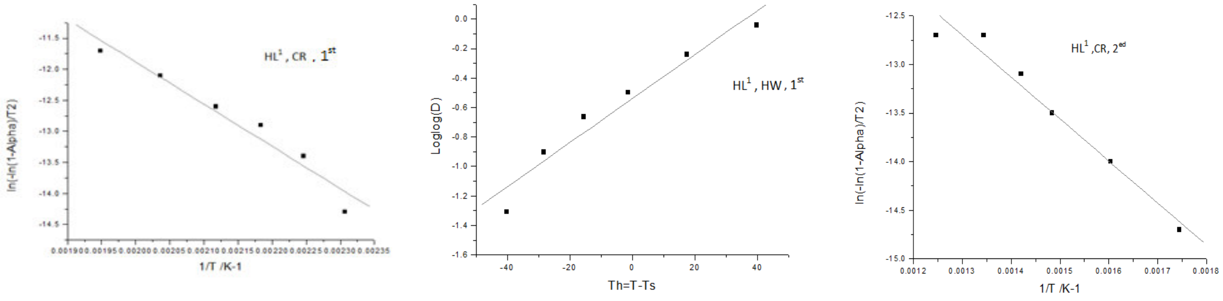

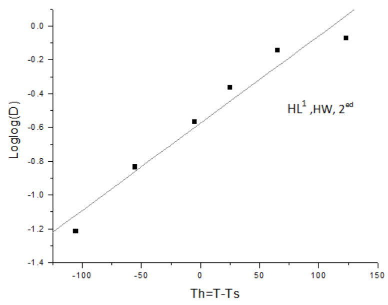
Loglog(D)
Th=T-Ts
HL^{1} ,HW, 2^{ed}

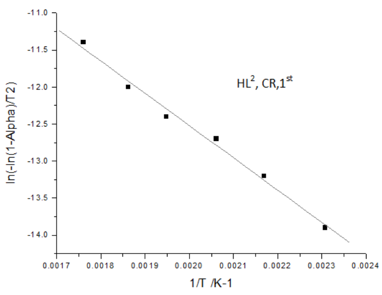
ln(-ln(1-Alpha)/T2)
1/T /K-1
HL^{2}, CR,1st

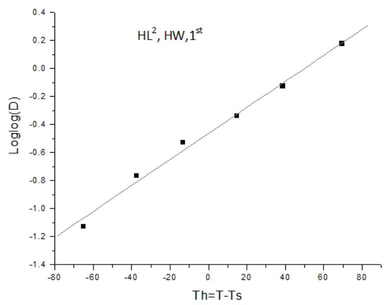
Loglog(D)
Th=T-Ts
HL^{2}, HW,1st

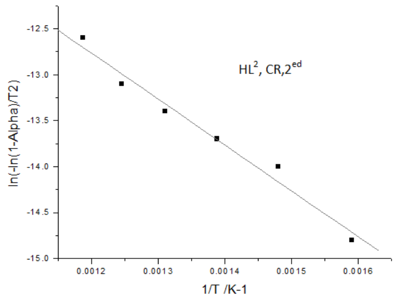
ln(-ln(1-Alpha)/T2)
1/T /K-1
HL^{2}, CR,2^{ed}

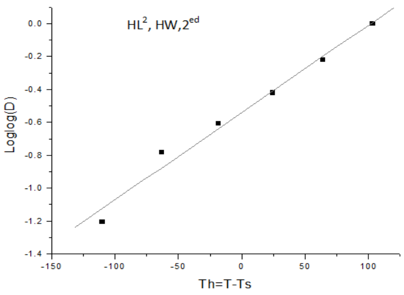
Loglog(D)
Th=T-Ts
HL^{2}, HW,2^{ed}

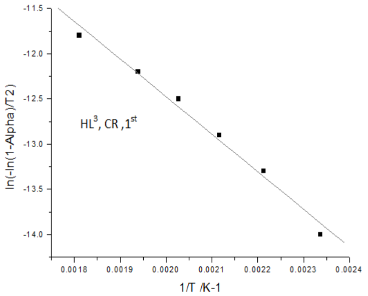
ln(-ln(1-Alpha)/T2)
1/T /K-1
HL^{3}, CR ,1st

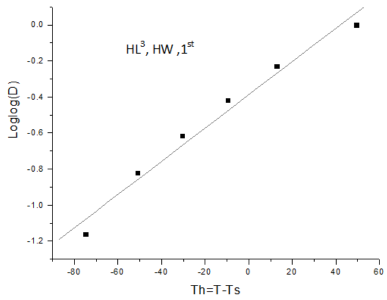
Loglog(D)
Th=T-Ts
HL^{3}, HW ,1st

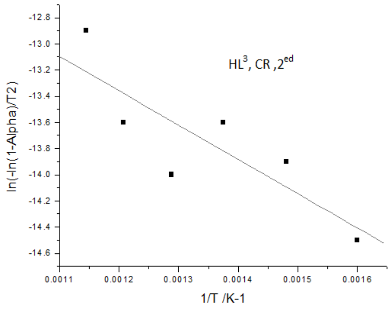
ln(-ln(1-Alpha)/T2)
1/T /K-1
HL^{3}, CR ,2^{ed}

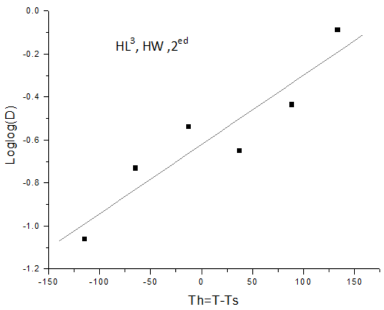
Loglog(D)
Th=T-Ts
HL^{3}, HW ,2^{ed}

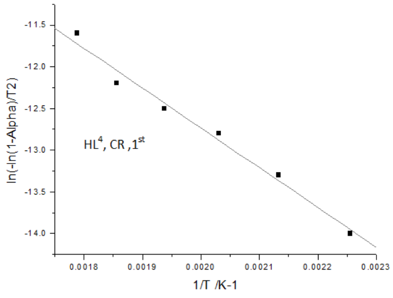
ln(-ln(1-Alpha)/T2)
1/T /K-1
HL^{4}, CR ,1st

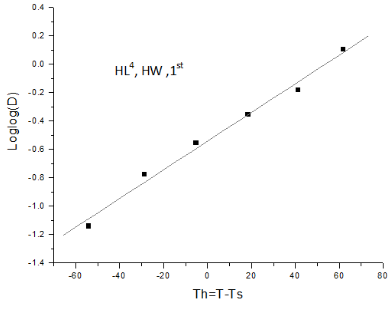
Loglog(D)
Th=T-Ts
HL^{4}, HW ,1st

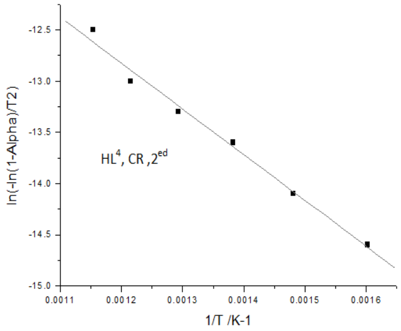
ln(-ln(1-Alpha)/T2)
1/T /K-1
HL^{4}, CR ,2^{ed}

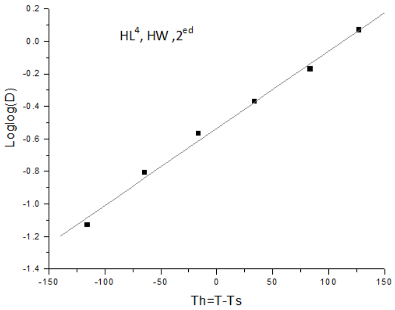

Figure 9A,S. kinetic plots using Coats Redfern and Horowitz-Metzger for pyrazolone liugands, HL(1-4)

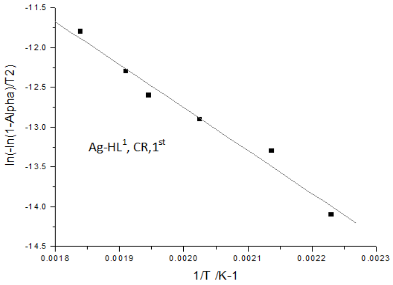

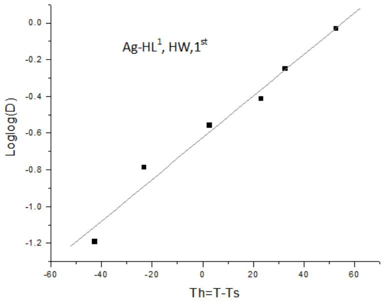

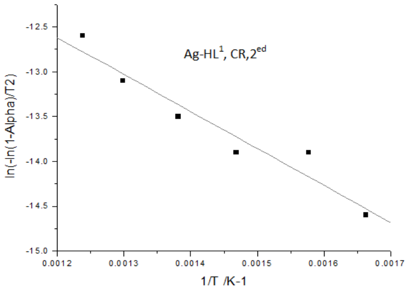

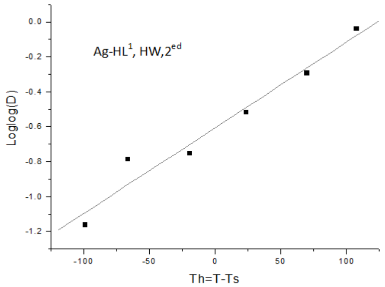

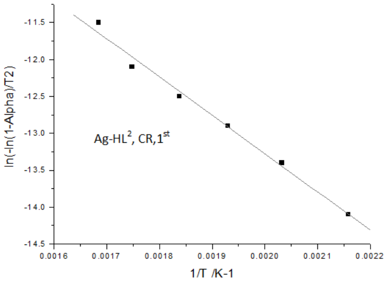

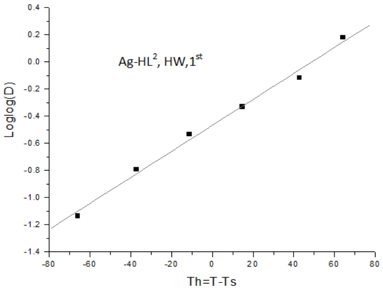

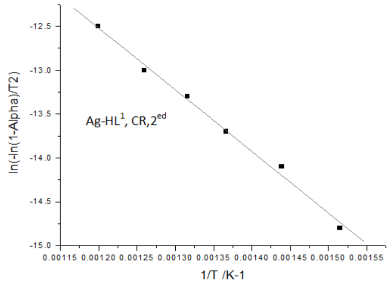

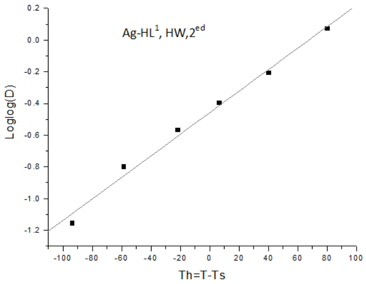

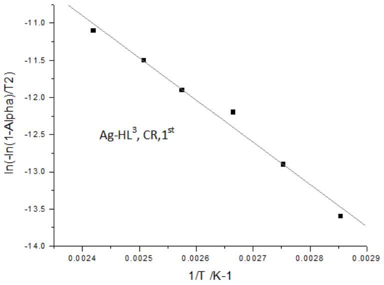

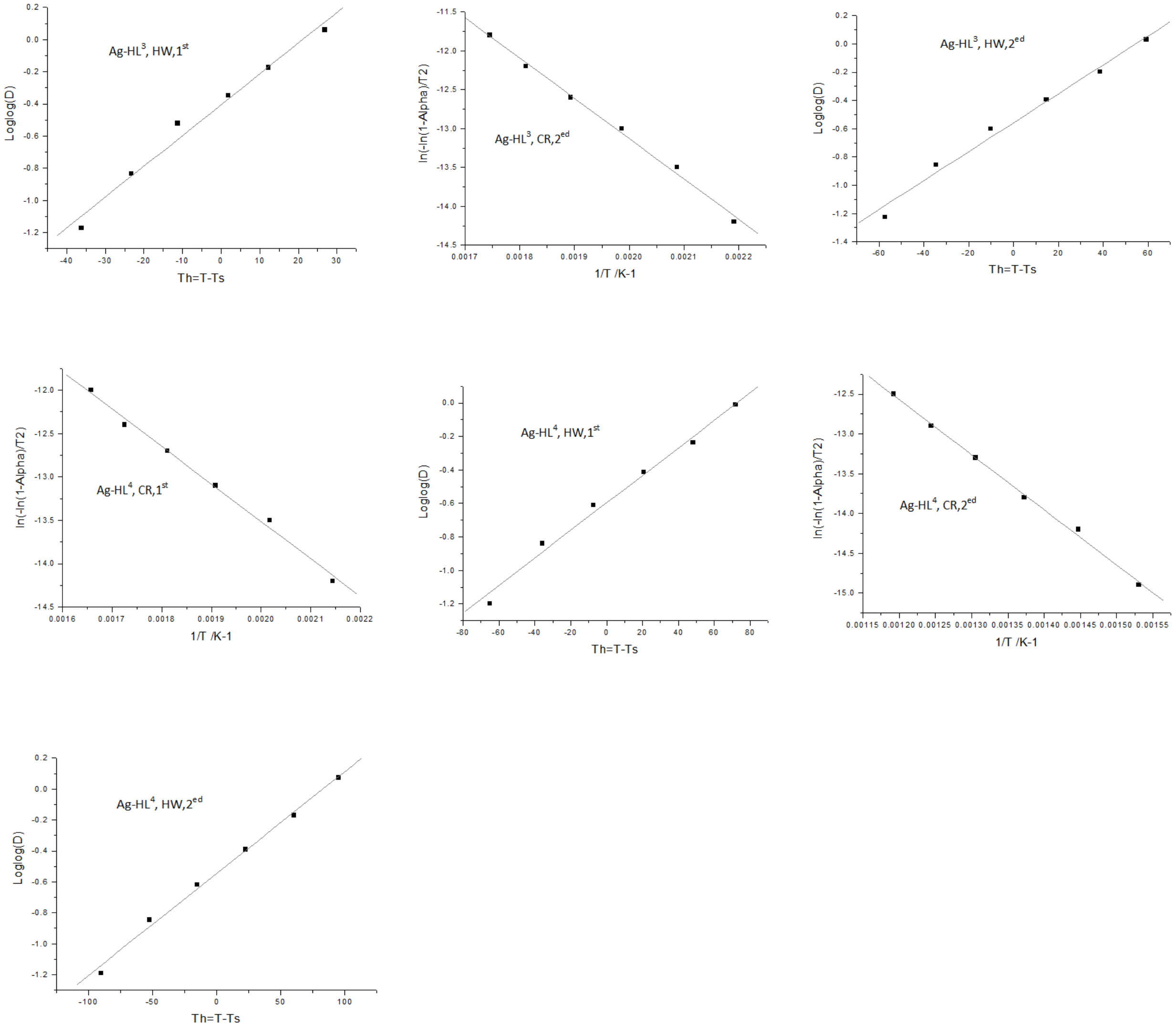

Figure 9B, S. kinetic plots using Coats Redfern and Horowitz-Metzger for pyrazolone- Ag(I) complexes

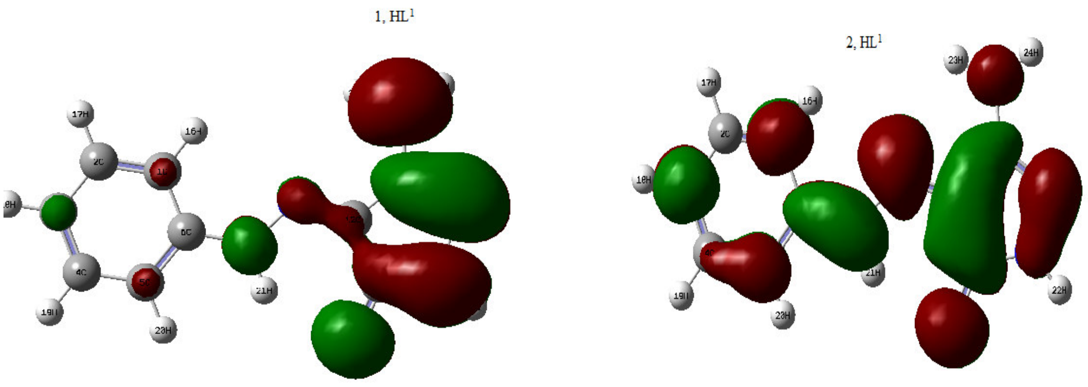

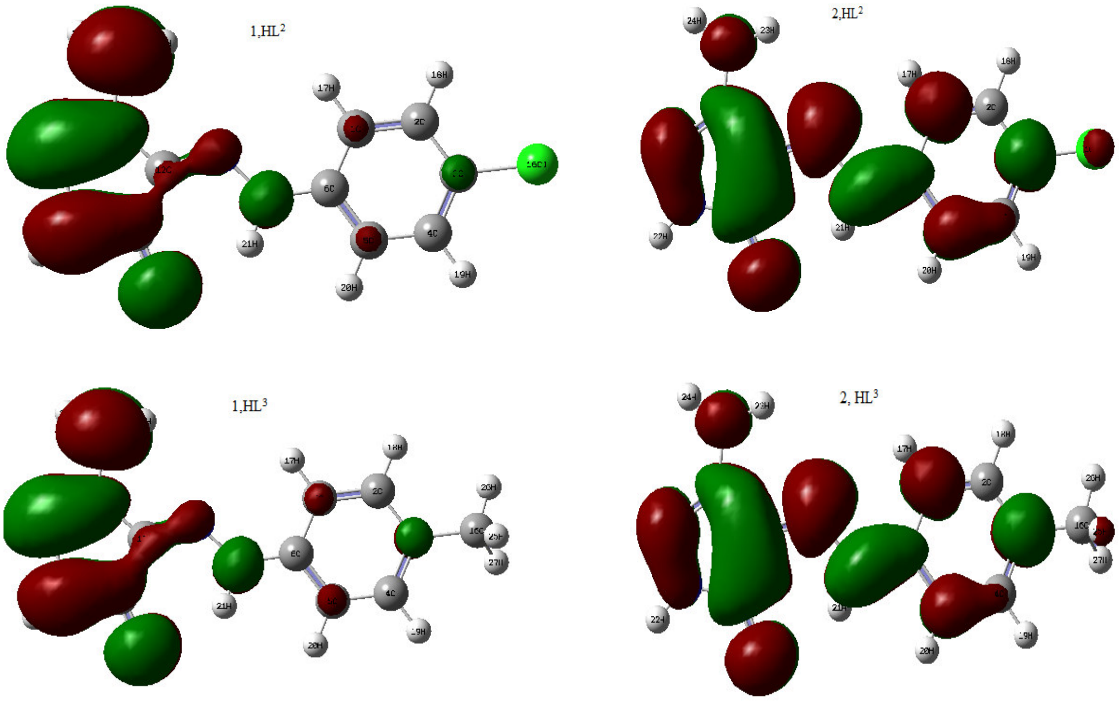

Figure 10A, S. Images of frontier molecular orbital's (HOMO,1 & LUMO,2) for pyrazolone ligands (HL $^{1-3}$)

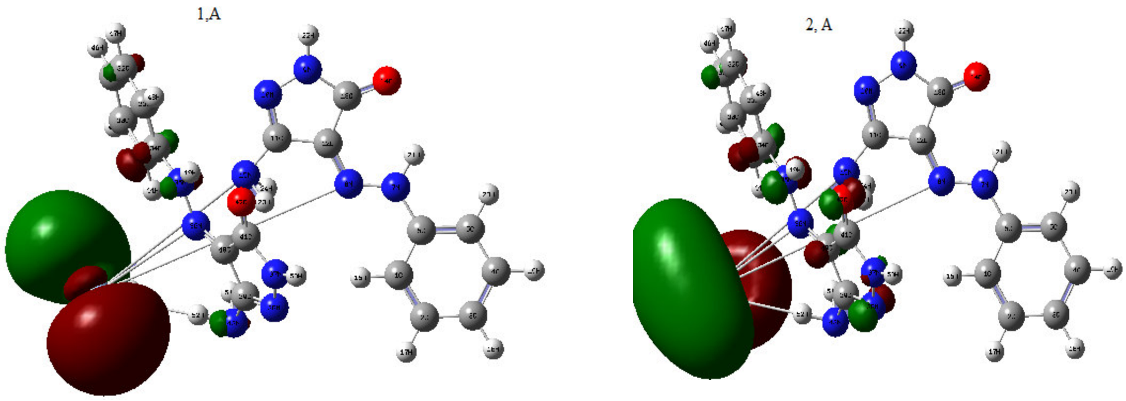

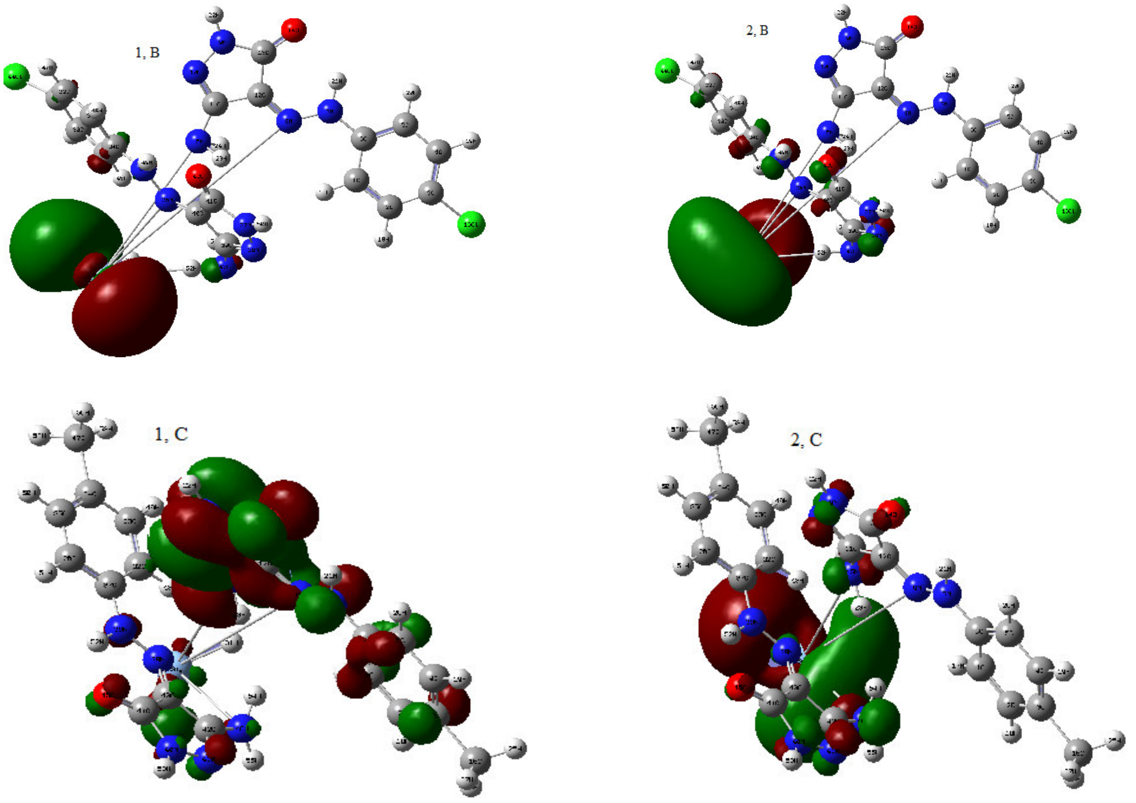

Figure 10B, S. Images of frontier molecular orbital's(HOMO,1 & LUMO,2) for Ag(I)-HL $^{1-3}$ complexes (A-c, respectively)

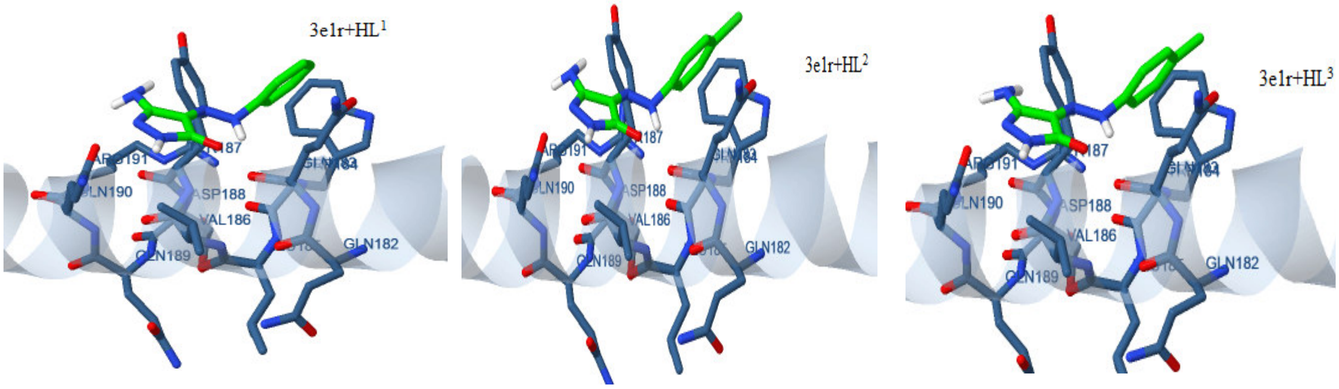

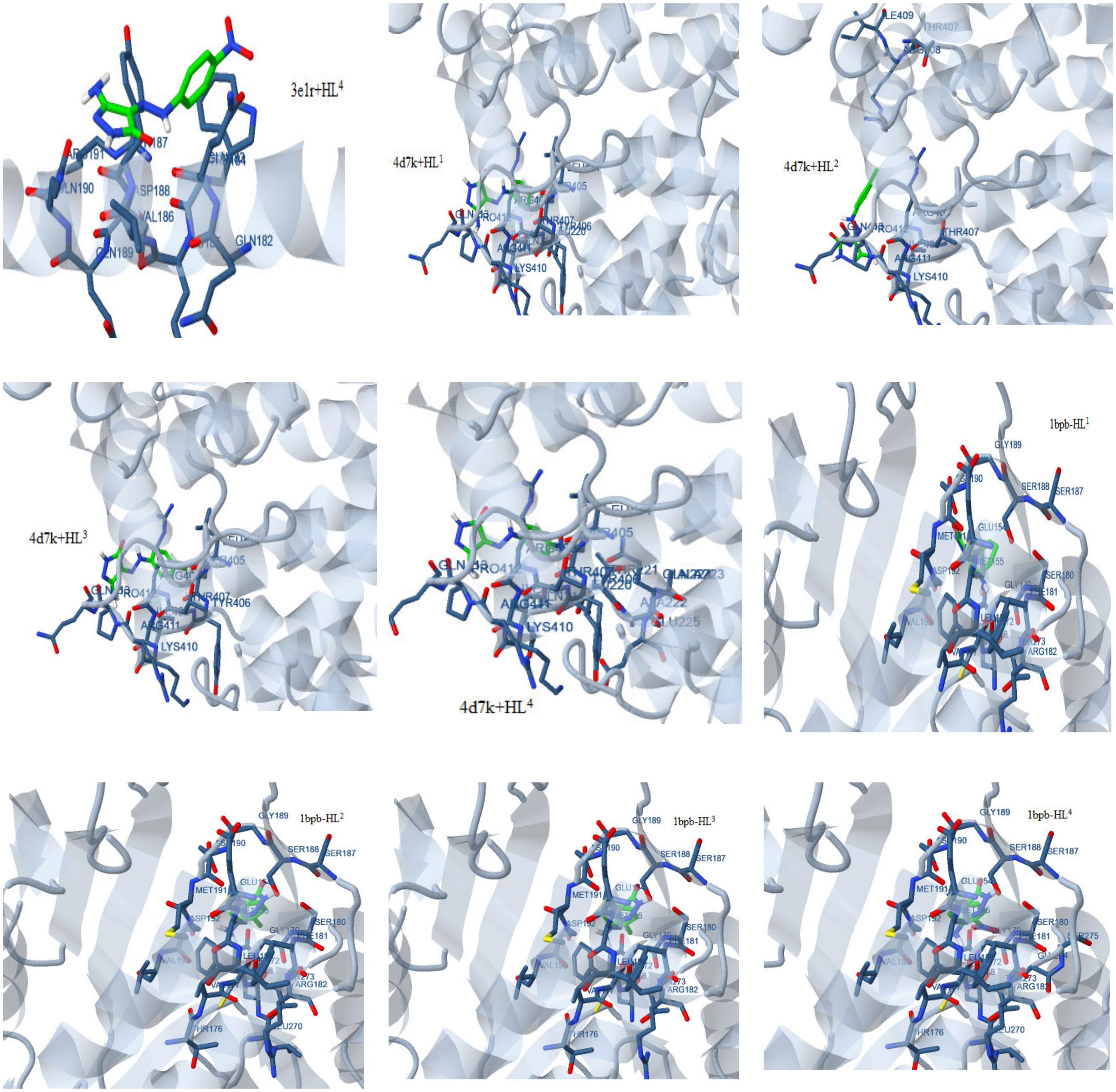

Figure 11**S**. Interacting complexes appeared with 3e1r , 4dk7 and 1bpb proteins and HL^{1-4} ligands

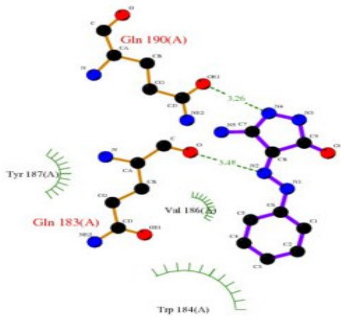

3e1r+HL[1]

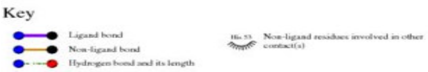

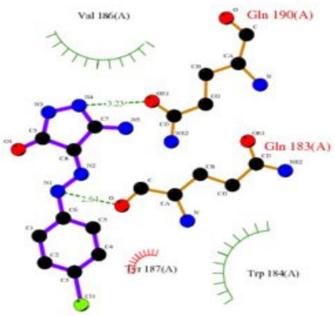

3e1r+HL[2]

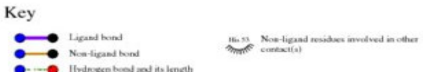

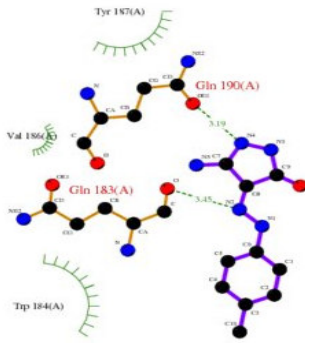

3e1r+HL[3]

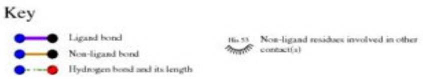

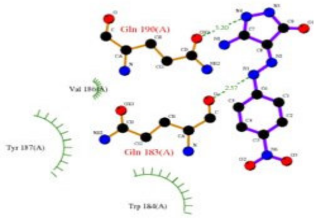

3e1r+HL[4]

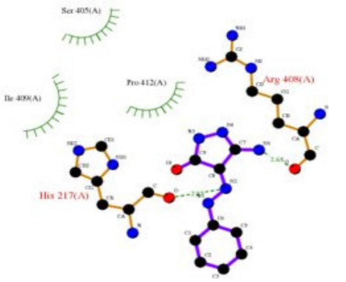

4d7k+HL[1]

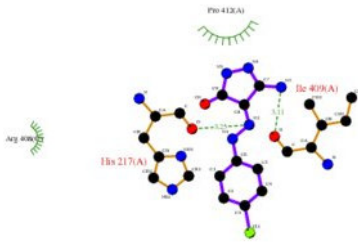

4d7k+HL[2]

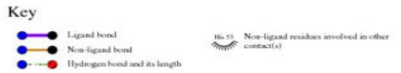

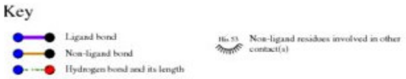

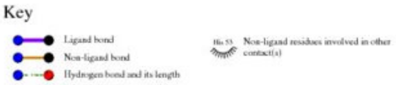

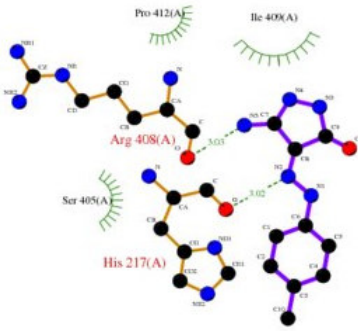

4d7k+HL[3]

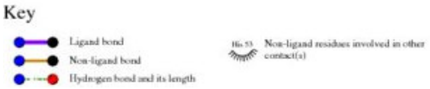

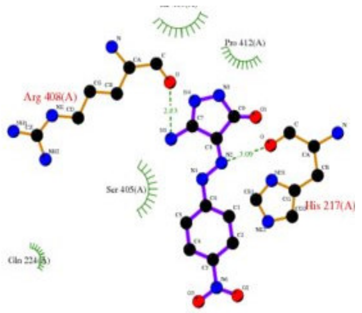

4d7k+HL[4]

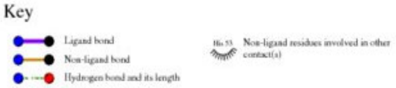

1bpb-HL[1]

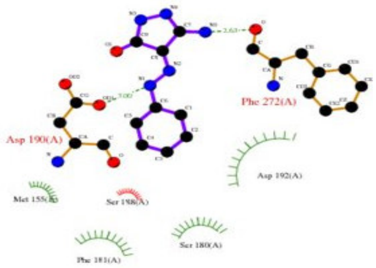

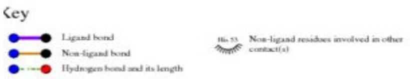

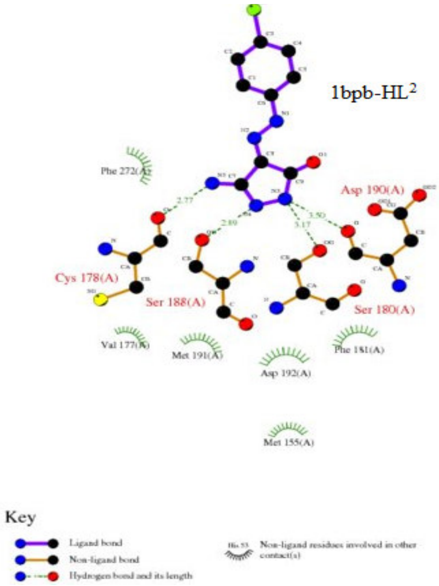

1bpb-HL^{2}

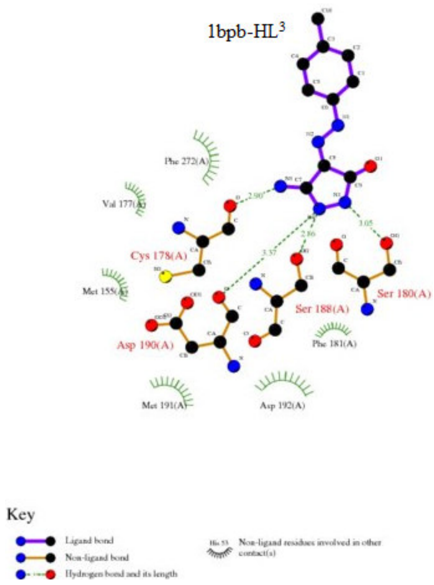

1bpb-HL^{3}

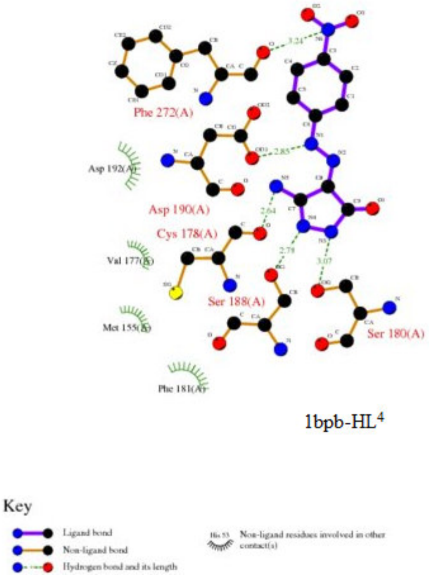

1bpb-HL^{4}

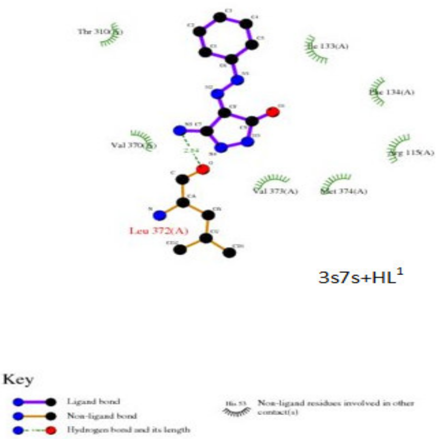

3s7s+HL^{1}

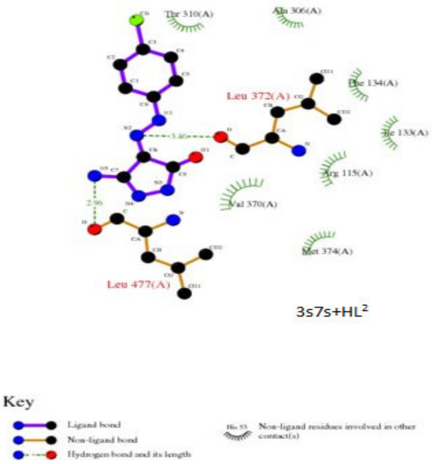

3s7s+HL^{2}

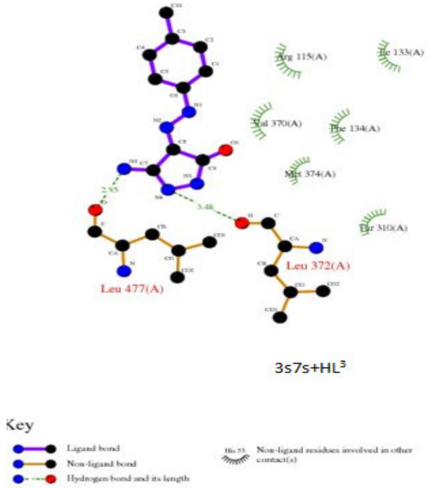

3s7s+HL^{3}

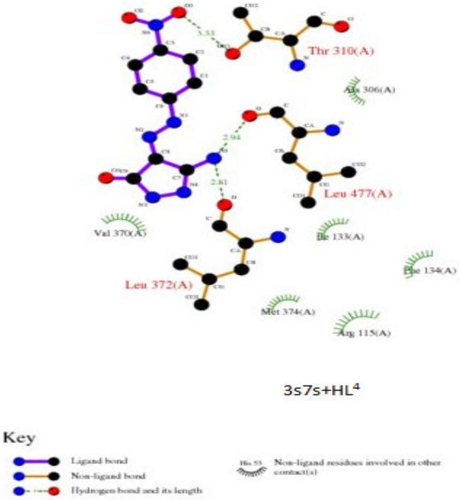

3s7s+HL^{4}

Figure 12S . 2D plots for interacting complexes appeared with 3e1r , 4dk7, 1bpb and 3s7s proteins and HL^{1-4} ligands
